# Supplementary material for: Association of self-reported snoring and hyperuricaemia: a large cross-sectional study in Chongqing, China
Source: BMJ Open. 2022 Apr 1;12(4):e056143. doi: 10.1136/bmjopen-2021-056143 (PMC8977806; doi:10.1136/bmjopen-2021-056143)
Supplement: Author's manuscript [file bmjopen-2021-056143.draft_revisions.pdf]

BMJ Open

BMJ Open is committed to open peer review. As part of this commitment we make the peer review history of every article we publish publicly available.

When an article is published we post the peer reviewers' comments and the authors' responses online. We also post the versions of the paper that were used during peer review. These are the versions that the peer review comments apply to.

The versions of the paper that follow are the versions that were submitted during the peer review process. They are not the versions of record or the final published versions. They should not be cited or distributed as the published version of this manuscript.

BMJ Open is an open access journal and the full, final, typeset and author-corrected version of record of the manuscript is available on our site with no access controls, subscription charges or pay-per-view fees (http://bmjopen.bmj.com).

If you have any questions on BMJ Open's open peer review process please email info.bmjopen@bmj.com

BMJ Open

Association of self-reported snoring and hyperuricemia: a large cross-sectional study in Chongqing, China

Journal:	*BMJ Open*
Manuscript ID	bmjopen-2021-056143
Article Type:	Original research
Date Submitted by the Author:	05-Aug-2021
Complete List of Authors:	chen, ting; Chongqing Medical University; Chongqing Municipal Center for Disease Control and Prevention<br>Tang, Wenge; Chongqing Municipal Center for Disease Control and Prevention<br>Chen, Liling; Chongqing Municipal Center for Disease Control and Prevention<br>Mao, Deqiang; Chongqing Municipal Center for Disease Control and Prevention<br>Song, Lingling; Chongqing Medical University, Department of Nutrition and Food Hygiene<br>Ding, Xianbin; Chongqing Municipal Center for Disease Control and Prevention<br>Lian, Xuemei; Chongqing Medical University, Department of Nutrition and Food Hygiene
Keywords:	EPIDEMIOLOGY, PREVENTIVE MEDICINE, PUBLIC HEALTH, Other metabolic, e.g. iron, porphyria < DIABETES & ENDOCRINOLOGY

SCHOLARONE™
Manuscripts

I, the Submitting Author has the right to grant and does grant on behalf of all authors of the Work (as defined in the below author licence), an exclusive licence and/or a non-exclusive licence for contributions from authors who are: i) UK Crown employees; ii) where BMJ has agreed a CC-BY licence shall apply, and/or iii) in accordance with the terms applicable for US Federal Government officers or employees acting as part of their official duties; on a worldwide, perpetual, irrevocable, royalty-free basis to BMJ Publishing Group Ltd ("BMJ") its licensees and where the relevant Journal is co-owned by BMJ to the co-owners of the Journal, to publish the Work in this journal and any other BMJ products and to exploit all rights, as set out in our licence.

The Submitting Author accepts and understands that any supply made under these terms is made by BMJ to the Submitting Author unless you are acting as an employee on behalf of your employer or a postgraduate student of an affiliated institution which is paying any applicable article publishing charge ("APC") for Open Access articles. Where the Submitting Author wishes to make the Work available on an Open Access basis (and intends to pay the relevant APC), the terms of reuse of such Open Access shall be governed by a Creative Commons licence – details of these licences and which Creative Commons licence will apply to this Work are set out in our licence referred to above.

Other than as permitted in any relevant BMJ Author's Self Archiving Policies, I confirm this Work has not been accepted for publication elsewhere, is not being considered for publication elsewhere and does not duplicate material already published. I confirm all authors consent to publication of this Work and authorise the granting of this licence.

For peer review only

Title: Association of self-reported snoring and hyperuricemia: a large cross-sectional study in Chongqing, China

Authors: Ting Chen[1,2], Wenge Tang[2], Liling Chen[2], Deqiang Mao[2], Lingling Song[1], Xianbin Ding[2*],Xuemei Lian[1*]

Affiliations:

1 Department of Nutrition and Food Hygiene, School of Public Health and Management, Chongqing Medical University, Chongqing, China

2 Chongqing Center for Disease Control and Prevention, Chongqing, China

*** Corresponding authors:**

Xuemei Lian, No.1 Yixueyuan Rd, Yuzhong District, Chongqing 400016, China, Email: xuemeilian@cqmu.edu.cn

Xianbin Ding, No.8 Changjiang 2 Rd, Yuzhong District, Chongqing 400042, China, Email: xianbinding@126.com

For peer review only

For peer review only - http://bmjopen.bmj.com/site/about/guidelines.xhtml

Abstract

Objective: To examine the relationship between self-reported snoring and hyperuricemia in a large-scale population in Chongqing, China.

Setting: Face-to-face electronic questionnaire survey, physical examination and biological sample testing were conducted in 13 districts Chongqing. Chongqing is a municipality in southwest China.

Participants: In this study, 23,308 Han ethnicity permanent residents aged 30 – 79 years were recruited. Individuals missing data were excluded, 22,389 subjects were included in final analysis.

Primary and secondary outcome measures: Serum uric acid was measured using an oxidase method. Hyperuricemia was defined as serum UA >420 μmol/L in men and >360 μmol/L in women. Information about self-reported snoring was obtained by questionnaire survey. All participants were divided into "no snoring" "snoring occasionally" and "snoring frequently". Multivariable logistic regression analysis was performed to assess the relationship between self-reported snoring and hyperuricemia.

Results: The prevalence of hyperuricemia was 14.43%, and snorers were more likely to have hyperuricemia than non-snorer in different age and gender groups. For the total population, those who snore occasionally or frequently were more likely to be hyperuricemia (OR 1.14, 95% CI 1.03-1.26; OR 1.23, 95% CI 1.11-1.37) compared with no snoring people. For the three age groups, those who snore frequently had a 24% increased risk of hyperuricemia (OR 1.24, 95% CI 1.05-1.48) compared with no snoring people in aged >59 group, but the association was not found in other two

For peer review only - http://bmjopen.bmj.com/site/about/guidelines.xhtml

groups. For male and female, those who snore frequently had a 15%, 28% increased risk of hyperuricemia than no snoring people in male and female group, respectively (OR 1.15, 95% CI 1.01-1.30; OR 1.28, 95% CI 1.07-1.52).

Conclusion: Snoring frequency was positively associated with higher risk of hyperuricemia, and the strength of association varied with different age and gender. We should pay attention to early intervention of snoring for preventing hyperuricemia.

Keywords: Self-reported snoring, Hyperuricemia, Chinese Han population

Strengths and limitations of this study:

The sample size of the study was relatively large.

This study analysis was thorough and comprehensive.

The cross-sectional data analysis limited ability to explore the casual relationship.

Our study results were limited by age and ethnicity.

Snoring frequency data was subjective.

Introduction

Hyperuricemia is a common chronic disease caused by purines metabolism dysfunction and uric acid excretion disorder. Hyperuricemia is becoming an important public health issue with its increasing prevalence in China. A meta-analysis study including 38 regional studies between 2000 and 2014 reported that the prevalence of hyperuricemia was 13.3% in mainland China [1]. Previous studies have confirmed that hyperuricemia not only is associated with the development of gout, but also increases risks of vascular diseases, diabetes mellitus, kidney disease, and metabolic syndrome [2-7]. Given the large disease burden related to hyperuricemia [8], it is necessary to

For peer review only

For peer review only - http://bmjopen.bmj.com/site/about/guidelines.xhtml

recognize more risk factors correlated with it.

Meanwhile, various major risk factors for hyperuricemia have been found, such as low economic status [9], obesity [10],and smoking [11], some studies have found a link between sleep-disordered breathing and hyperuricemia [12-17]. However, the association of snoring and hyperuricemia has been studied much less extensively. To our knowledge, only three studies [12 15 16] have been referred to explore the relationship of snoring and hyperuricemia. In addition, the three studies had small sample sizes, considered confounding factors insufficiently and did not examine the association by age stratified analysis.

This study aimed to examine comprehensively the relationship of self-reported snoring and hyperuricemia after controlling for potential confounding factors in a large-scale population in Chongqing, China.

Methods

Participants

The present study was from the baseline survey of the China Multi-Ethnic Cohort Study [18], Chongqing region, which is a collaborated study by Sichuan University, Chongqing Medical University and Chongqing Center for Disease Control and Prevention. This study was carried out in 13 districts Chongqing, including Yuzhong District, Jiulongpo District, Nan·an District, Ba·nan District, Changshou District, Jiangjin District, Hechuan District, Qijiang District, Dazu District, Tongnan District, Rongchang District, Wulong District, Fengdu District. In brief, a face-to-face electronic questionnaire survey, physical examination and biological sample testing

For peer review only - http://bmjopen.bmj.com/site/about/guidelines.xhtml

were conducted in this study. In order to ensure the quality of research, some quality control measures were applied, for example checking electronic recording. A total of 23,308 Han ethnicity permanent residents aged 30 – 79 years were recruited by using randomized sampling during September 2018 to January 2019. The sample inclusion criteria and exclusion criteria have been described in detail previously [18]. We excluded individual data missing serum uric acid (n=859) or snoring (n=14) or blood pressure (n=18) or blood lipid level (n=28). Finally, this present study consisted of 22,389 (figure 1) individuals providing complete information. All participants must written informed consent prior to the study. This study was approved by the ethics committee of Sichuan University (No.K2016038).

Measures

Serum uric acid (UA) measurement and the definition of hyperuricemia

After fasting for 12 hours, venous blood samples were collected from all participants. Within 2 hours after collection, blood samples were transported in dry ice to one medical laboratory certified by Chongqing Center for Disease Control and Prevention. Serum UA was measured with a Beckman Coulter AU 680 device using an oxidase method in Chongqing Di·an Medical Laboratory Center. The validity and accuracy of UA were 10% and 4%, respectively. Hyperuricemia was defined as serum UA >420 μmol/L in men and >360 μmol/L in women [9 19].

Measurement of snoring

Information about self-reported snoring was obtained by the question "Do you snore when you have a sleep?" The frequency of snoring ("occasionally" and "frequently")

For peer review only - http://bmjopen.bmj.com/site/about/guidelines.xhtml

was asked for those who answered "yes". Finally, all participants were divided into "no snoring" "snoring occasionally" and "snoring frequently".

Assessment of covariates

Sociodemographic characteristics included in our study age, gender (male, female), marital status, education level, residential region (urban, rural), employment status (yes, no), household yearly income level (＜60,000 RMB,⩾60,000 RMB). Consistent with previous studies in china [20 21], participates were grouped into low-aged adults (＜45 years), middle-aged adults (45-59 years) and older adults (＞59 years). Marital status was categorized into married or living with partner and others, including widowed, unmarried, divorced or separated. Education level was classified into primary or below (elementary school or illiterate), secondary (junior school, senior school or secondary vocational school) and college or above.

Moreover, the present study collected information about many health-related behaviors. Smoking status was grouped into never smoker, current smoker and ever smoker. Alcohol drinking status was categorized into never or hardly drinker, occasionally drinker (only drink on special occasions or the frequency less than once a week), often drinker (at least once a week in the past year). Tea drinking status was defined by the question "Have you ever been drinking tea weekly for more than half a year?" Accordingly, participants were divided into tea drinker and non-tea drinker. Oil and salt intake were collected by asking about the consumption of oil and salt per month in the past year. Energy intake was measured using a semi-quantitative standardized food frequency questionnaire with 13 food categories. Physical activity

For peer review only - http://bmjopen.bmj.com/site/about/guidelines.xhtml

level (expressed as metabolic equivalent, MET) was estimated based on self-reported activities using a Compendium of Physical Activities [22], and the physical activities questionnaire referenced the global physical activity questionnaire. Nocturnal sleep and middy nap duration were collected according to the two questions: "how many hours do you usually nocturnal sleep every day in the past month ?" and "how many minutes do you usually middy nap every day in the past month ?"

Lastly, the information regarding clinic variables were obtained including body mass index (BMI), dyslipidemia, diabetes and hypertension. BMI was calculated as weight divided by height^2 (kg/m^2). Dyslipidemia was defined using serum total cholesterol concentration of ⩾6.2mmol/l, triacylglycerol concentration of ⩾2.3 mmol/l, low-density lipoprotein cholesterol concentration of ⩾4.1 mmol/l, high-density lipoprotein cholesterol concentration of < 1.0 mmol/l or a self-reported diagnosis of dyslipidemia[23]. Diabetes was diagnosed as fasting blood glucose level of ⩾7.0 mmol/l, glycosylated hemoglobin percentage of ⩾6.5% or a self-reported diagnosis of diabetes[24]. Participants with were regarded as having hypertension if any of the following three items were met: systolic blood pressure of ⩾140 mmHg, diastolic blood pressure of ⩾ 90 mmHg or self-reported diagnosis of hypertension[25].

Statistical analysis

The analyses were performed with the SPSS, version 25.0. Characteristics of the participants, including sociodemographic characteristics, health-related behaviors and clinic variables, were compared according to the frequency of snoring ("no", "occasionally" and "frequently"). All continuous data were described as mean±

For peer review only - http://bmjopen.bmj.com/site/about/guidelines.xhtml

standard deviations, while categorical data were summarized as percentages. The one-way analysis of variance was used to compare the difference in continuous data, and chi-square test was used to compare the difference in categorical data. Then, the prevalence of hyperuricemia with different snoring frequency was examined using the chi-square test in different age and gender groups. Finally, Multivariable logistic regression analysis was performed to evaluate the relationship between snoring and hyperuricemia after adjusting for different covariates in different age groups, gender groups and total population, and odds ratio (OR) and 95% confidence interval (CI) for the outcome variable were calculated. Two-sided *p value of* <0.05 was considered to be statistically significant.

Patient and public involvement

Patients and the public were not involved in the design of the study, recruitment and conduct of the study or dissemination of the study results.

Results

Sample characteristics of participants

The characteristics of participants based on the categories of self-reported snoring are showed in Table 1.The study composed of 22,389 individuals in the final analysis. Of the study subjects, 51.23%, 26.71%, 22.06% reported “no snoring”, “snoring occasionally”, “snoring frequently”, respectively. Participants who snore were older and more likely to be male, unemployed person, smokers, alcohol drinkers, tea drinkers. In addition, compared with those who reported “no snoring”, snorers had a higher level of energy take, middy nap duration, BMI, dyslipidemia, diabetes,

For peer review only - http://bmjopen.bmj.com/site/about/guidelines.xhtml

hypertension, but had a lower level of physical activity, nocturnal sleep duration.

Table 1 Characteristics of participants based on the categories of self-reported snoring

**Characteristics**	**Self-reported snoring**			***P* value**
	**No**	**Occasionally**	**Frequently**	
Number of subjects	11469	5981	4939	
**Sociodemographic Characteristics**				
Age				< 0.001
< 45	36.18	30.16	19.80	
45-59	39.64	42.37	43.59	
> 59	24.18	27.47	36.61	
Male	35.26	53.02	66.13	< 0.001
Married or living with partner	87.75	88.13	88.01	0.742
Education level				< 0.001
Primary or below	33.16	28.57	36.83	
Secondary	49.94	53.10	49.20	
College or above	16.90	18.32	13.97	
Urban	27.49	33.42	25.81	< 0.001
Employment	63.05	62.43	59.73	
Income (< 60,000 RMB)	59.51	55.12	59.91	< 0.001
**Health-related behaviors**				
Smoking				< 0.001
Never	80.99	69.27	59.71	
Current	14.61	23.61	31.00	
Ever	4.40	7.12	9.29	
Alcohol drinking				< 0.001
Never or hardly	53.00	38.40	37.84	
Occasionally	37.56	45.23	40.27	
Often	9.44	16.37	21.89	

For peer review only

For peer review only - http://bmjopen.bmj.com/site/about/guidelines.xhtml

Tea drinker	16.27	24.33	28.10	< 0.001
Oil intake (g/week)	56.71±0.30	56.66±0.42	58.33+0.47	0.008
Salt intake (g/week)	48.36±0.27	47.45±0.36	51.04±0.42	< 0.001
Energy intake (kcal/week)	12776.57±39.74	13241.66±56.36	13838.48±64.34	< 0.001
Physical activity (MET minutes /week)	10176.77±63.98	9731.86±86.57	9777.99±101.00	< 0.001
Nocturnal sleep duration (h/day)	7.18±0.01	7.04±0.02	6.97±0.02	< 0.001
Middy nap duration (min/day)	28.91±0.33	31.17±0.45	33.43±0.54	< 0.001
**Clinic variables**				
BMI ((kg/m^2))	23.83±0.03	24.93±0.04	26.26±0.05	< 0.001
Dyslipidemia	23.99	33.46	44.26	< 0.001
Diabetes	7.35	10.18	15.02	< 0.001
Hypertension	28.13	36.23	50.50	< 0.001

Continuous data were described as mean± standard deviations, and statistical significance was assessed by the one-way analysis of variance.

Categorical data were summarized as percentages (%), and statistical significance was assessed by chi-square test.

The prevalence of hyperuricemia

The prevalence of hyperuricemia according to different snoring frequency with different groups is presented in Table 2. In all, the prevalence of hyperuricemia was 14.43% (3230/22389) in our study population. Compared with the “no snoring” group, the prevalence of hyperuricemia was higher in the “snoring occasionally” and “snoring frequently” groups among the three age groups (P<0.05). In addition, those who reported “snoring occasionally” or “snoring frequently” were more likely to have hyperuricemia than “no snoring” group in different gender groups (P<0.05). Similarly, in the total population, higher prevalence of hyperuricemia was found in the “snoring

For peer review only - http://bmjopen.bmj.com/site/about/guidelines.xhtml

occasionally" or "snoring frequently" compared with "no snoring" group.

Table 2 Prevalence of hyperuricemia according to snoring frequency in different groups

Snoring frequency	Age [n (%)]			Gender [n (%)]		Total
	< 45	45-59	> 59	Male	Female	
No	376(9.06)	441(9.70)	358(12.91)	650(16.07)	525(7.07)	1175(10.25)
Occasionally	326(18.07)	370(14.60)	261(15.89)	659(20.78)	298(10.60)	957(16.00)
Frequently	299(30.57)	452(20.99)	347(19.19)	820(25.11)	278(16.62)	1098(22.23)
$\chi 2$	322.34	160.32	33.23	91.72	156.38	418.14
P value	< 0.001	< 0.001	< 0.001	< 0.001	< 0.001	< 0.001

Association self-reported snoring and hyperuricemia

Results of multivariable logistic regression analysis about the association self-reported snoring and hyperuricemia are showed in Table 3. In the total population, after adjusting for sociodemographic characteristics, health-related behaviors and clinic variables, those who reported "snoring occasionally" or "snoring frequently" were more likely to be hyperuricemia (OR 1.14, 95%CI 1.03-1.26; OR 1.23, 95%CI 1.11-1.37, respectively) compared with "no snoring" group. The stratified analysis by age results showed: after adjusting for all variables, higher odd ratio of hyperuricemia (OR 1.24, 95%CI 1.05-1.48) was found in the "snoring frequently" group compared with "no snoring" group in aged >59 group, but the association was not found in aged 45- 59 and aged <45 groups (P>0.05, respectively). The stratified analysis by gender results showed: after adjusting for all variables, those who reported "snoring frequently" had a higher odd ratio of hyperuricemia in male (OR 1.15, 95%CI 1.01-1.30) and female (OR 1.28, 95%CI 1.07-1.52, respectively) than "no snoring"

For peer review only - http://bmjopen.bmj.com/site/about/guidelines.xhtml

group.

Table 3 Association self-reported snoring and hyperuricemia

Variables	NO	Occasionally		Frequently	
	OR（95%CI）	OR（95%CI）	*P* value	OR（95%CI）	*P* value
**Age**					
**＜45**					
Model 1	1.00(reference)	2.21(1.89,2.60)	＜0.001	4.42(3.72,5.25)	＜0.001
Model 2	1.00(reference)	1.51(1.28,1.79)	＜0.001	2.44(2.03,2.94)	＜0.001
Model 3	1.00(reference)	1.49(1.25,1.77)	＜0.001	2.39(1.97,2.88)	＜0.001
Model 4	1.00(reference)	1.11(0.93,1.34)	0.25	1.18(0.96,1.46)	0.13
**45-59**					
Model 1	1.00(reference)	1.59(1.37,1.85)	＜0.001	2.47(2.14,2.85)	＜0.001
Model 2	1.00(reference)	1.33(1.14,1.55)	＜0.001	1.88(1.62,2.18)	＜0.001
Model 3	1.00(reference)	1.29(1.11,1.51)	0.00	1.84(1.58,2.14)	＜0.001
Model 4	1.00(reference)	1.06(0.91,1.25)	0.45	1.17(1.00,1.38)	0.06
**＞59**					
Model 1	1.00(reference)	1.28(1.07,1.52)	0.01	1.60(1.36,1.88)	＜0.001
Model 2	1.00(reference)	1.25(1.05,1.48)	0.01	1.59(1.35,1.87)	＜0.001
Model 3	1.00(reference)	1.22(1.03,1.46)	0.03	1.58(1.34,1.86)	＜0.001
Model 4	1.00(reference)	1.11(0.93,1.33)	0.24	**1.24(1.05,1.48)**	**0.01**
**Gender**					
**Male**					
Model 5	1.00(reference)	1.37(1.22,1.54)	＜0.001	1.75(1.56,1.96)	＜0.001
Model 6	1.00(reference)	1.30(1.15,1.47)	＜0.001	1.79(1.59,2.00)	＜0.001
Model 7	1.00(reference)	1.25(1.11,1.41)	＜0.001	1.72(1.53,1.93)	＜0.001
Model 8	1.00(reference)	1.05(0.92,1.19)	0.50	**1.15(1.01,1.30)**	**0.04**

For peer review only

**Female**					
Model 5	1.00(reference)	1.56(1.34,1.81)	< 0.001	2.62(2.24,3.06)	< 0.001
Model 6	1.00(reference)	1.42(1.22,1.65)	< 0.001	2.13(1.81,2.50)	< 0.001
Model 7	1.00(reference)	1.42(1.22,1.65)	< 0.001	2.12(1.80,2.50)	< 0.001
Model 8	1.00(reference)	1.16(0.99,1.35)	0.07	**1.28(1.07,1.52)**	**0.01**
**Total**					
Model 9	1.00(reference)	1.67(1.52,1.83)	< 0.001	2.50(2.29,2.74)	< 0.001
Model 10	1.00(reference)	1.45(1.32,1.59)	< 0.001	2.03(1.84,2.23)	< 0.001
Model 11	1.00(reference)	1.43(1.30,1.57)	< 0.001	2.00(1.82,2.20)	< 0.001
Model 12	1.00(reference)	**1.14(1.03,1.26)**	**0.01**	**1.23(1.11,1.37)**	**< 0.001**

Model 1: unadjusted model

Model 2: adjusted for gender, marital status, education level, residential region, employment status and household yearly income level

Model 3: additionally adjusted for smoking status, alcohol drinking status, tea drinking status, oil intake, salt intake, energy intake, physical activity level, nocturnal sleep and middy nap duration

Model 4: additionally BMI, dyslipidemia, diabetes and hypertension

Model 5: unadjusted model

Model 6: adjusted for age, marital status, education level, residential region, employment status and household yearly income level

Model 7: additionally adjusted for smoking status, alcohol drinking status, tea drinking status, oil intake, salt intake, energy intake, physical activity level, nocturnal sleep and middy nap duration

Model 8: additionally adjusted for BMI, dyslipidemia, diabetes and hypertension

Model 9: unadjusted model

Model 10: additionally adjusted for age, gender, marital status, education level, residential region, employment status and household yearly income level

Model 11: additionally adjusted for smoking status, alcohol drinking status, tea drinking status, oil intake, salt intake, energy intake, physical activity level, nocturnal sleep and middy nap duration

Model 12: additionally adjusted for BMI, dyslipidemia, diabetes and hypertension

Discussion

In our study, we found that self-reported snoring was independently associated with hyperuricemia in total population. After adjusting for the sociodemographic characteristics, health-related behaviors and clinic variables, those

For peer review only - http://bmjopen.bmj.com/site/about/guidelines.xhtml

For peer review only

who reported "snoring occasionally" and "snoring frequently" had a 14% and 23% increased risk of hyperuricemia compared with "no snoring" group.

Our findings are consistent with those results of previous studies. An analysis of 6491 participants from the National Health and Nutrition Survey 2005–2008 found an increased risk of hyperuricemia associated with snoring, which found similar results with our study[15]. Xiaolu et al. enrolled 7699 Chinese urban adults from Nanjing between September 2016 and February 2018, they verified the positive link between snoring and hyperuricemia [16]. A study of data from the baseline survey of the China Hainan Centenarian Cohort Study (CHCCS) also showed a positive association between snoring and hyperuricemia in centenarians [12]. The possible mechanisms can partly explain the positive association between snoring and hyperuricemia. Snoring is often accompanied by oxygen desaturation leading to tissue hypoxia, and then this accelerates the breakdown of adenosine triphosphate, resulting in the accumulation of uric acid from purine catabolic product by xanthine dehydrogenase and xanthine oxidase [26]. In addition, some studies have showed that snoring is related to the inflammation response by vibration tissue cell releasing inflammatory factor [27 28], and previous study have found the association of inflammation response and metabolic disease, for example dyslipidemia [29 30]. Therefore, whether the effect of snoring on hyperuricemia is mediated by inflammatory pathway needs to be further exploration.

Compared with previous studies, our study conducted a more detailed relationship analysis of snoring and hyperuricemia in different age and gender groups. To our knowledge, no studies have been analyzed age differences of association between

For peer review only - http://bmjopen.bmj.com/site/about/guidelines.xhtml

snoring and hyperuricemia. We found that those who snore frequently with aged >59 year old had a 24% increased risk of hyperuricemia ($P<0.05$) compared with no snoring people, however, the positive association did not found in the other two age groups. This result suggested that the association between snoring frequency and hyperuricemia may vary with age. In the present study, for male and female, those who snore frequently had a 15% and 28% increased risk of hyperuricemia ($P<0.05$) than no snoring people, respectively. Our findings showed the strength of association in female was stronger compared with male, which are consistent with previous study [16]. To the best of our knowledge, no study has explored the possible mechanisms. It might be related to hormones difference in female and male. Therefore, more evidence studies on the mechanism of different strength relationship between snoring and hyperuricemia in female and male are needed. In this study, our results showed snoring occasionally and snoring frequently had increased risk of hyperuricemia than no snoring people in all different age and gender groups before adjusting for clinic variables, although the association between snoring occasionally and no snoring was not found for all group after adjusting for all confounders. This implies that BMI, dyslipidemia, diabetes and hypertension may affect the relationship between snoring frequency and hyperuricemia in different age and gender groups. Previous studies had confirmed snoring to be significantly association with obesity [31], dyslipidemia [32], diabetes [33] and hypertension [34]. Therefore, it is necessary to adjust clinical indicators.

In addition, our results showed that the prevalence of hyperuricemia was 14.43% in Chongqing, which was similar to the results of the Tianjin Brain Study (14.4%) [35].

For peer review only

For peer review only - http://bmjopen.bmj.com/site/about/guidelines.xhtml

However, this prevalence was more than that in the Tangshan population (13.4%) [36]. The difference may be explained by the different lifestyles and economic level in the two different areas, which are also closely related to hyperuricemia [37]. Our determined prevalence was also significantly higher than that in china national hyperuricemia survey, which showed that the prevalence of hyperuricemia was 8.4% in 2009–2010 [38]. This indicates that we should pay more attention to hyperuricemia due to the prevalence is increasing with the improvement of economic level and lifestyles changes.

Compared with previous study, our study is more thorough and comprehensive because the stratified analysis was conducted to explore the difference of relationship between snoring and hyperuricemia in different age and gender groups. Only one previous study conducted a stratified analysis by gender, but this study did not consider to stratified analysis by age and the sample population of this study was not representative due to the subjects from hospital [16]. To the best of our knowledge, this is the first study to explore the association between snoring and hyperuricemia used a large sample size in Southwest china. Furthermore, the present study included many potential confounders, such as smoking, alcohol drinking, tea drinking, oil take, salt take, energy take, physical activity, sleep duration, nap duration, BMI, dyslipidemia, diabetes and hypertension, which could minimize the bias.

The present study also has some limitations. First, our study data was a cross-sectional result from the cohort study, and the cross-sectional data analysis limited ability to explore the casual relationship between snoring and hyperuricemia.

For peer review only - http://bmjopen.bmj.com/site/about/guidelines.xhtml

Further follow-up data analysis could confirm whether there was causal relationship between snoring and hyperuricemia by using special statistical model. Second, snoring frequency was obtained from a subjective self-reported question, which may be different with actual snoring frequency. However, Jennum et al. had found the self-reported snoring frequency was highly significant related to snoring record by microphone objectively [39], suggesting that the self-reported snoring could be applied in scientific research. Third, the present study was lack of degree of snoring, which may distort the relationship between snoring and hyperuricemia. Thus, the degree of snoring should be considered in future studies. Fourth， our study was limited by age and ethnicity, so extrapolating the results to other age groups and ethnicity population should be noted.

Conclusions

In summary, snoring frequency was positively associated with higher risk of hyperuricemia in total population. In aged >59 population, those who snore frequently had increased risk of hyperuricemia compared with no snoring people, but the association was not found in aged 45- 59 and aged <45 groups. In addition, snore frequently is independently risk factor of hyperuricemia for male and female, and the strength of association in female was stronger compared with male. Our results indicated that early intervention of snoring may help to prevent hyperuricemia.

Acknowledgements

We really appreciate all participants, project staff of the China Multi-Ethnic Cohort Study.

For peer review only - http://bmjopen.bmj.com/site/about/guidelines.xhtml

Contributions

All authors contributed significantly to this article.TC analyzed the data and wrote the first version of the manuscript; WT, LC, DM and XD performed the surveys; LS and XL interpreted the results and revised the manuscript; XD and XL critically reviewed the manuscript for important intellectual content. All authors approved the final manuscript.

Funding

This work was supported by National Key R&D Program of China (grant number: 2017YFC0907303) and Chongqing Health branding Subject Fund (grant number: 2020FYYX013).

Competing interests

None declared.

Patient consent

Obtained.

Ethics approval

The study was approved by the ethics committee of Sichuan University (No.K2016038).

Provenance and peer review

Not commissioned; externally peer reviewed.

Availability of data and materials

Our study relied on data from China Multi-Ethnic Cohort Study. The summary dataset used and or analyzed during the current study are available from the corresponding

For peer review only

author on a reasonable request.

References

1. Liu R, Han C, Wu D, et al. Prevalence of Hyperuricemia and Gout in Mainland China from 2000 to 2014: A Systematic Review and Meta-Analysis. *Biomed Res Int* 2015;2015:762820.
2. Chen JH, Chuang SY, Chen HJ, et al. Serum uric acid level as an independent risk factor for all-cause, cardiovascular, and ischemic stroke mortality: a Chinese cohort study. *Arthritis Rheum* 2009;61:225-32.
3. Dehghan A, van Hoek M, Sijbrands EJ, et al. High serum uric acid as a novel risk factor for type 2 diabetes. *Diabetes Care* 2008;31:361-2.
4. Fang J, Alderman MH. Serum uric acid and cardiovascular mortality the NHANES I epidemiologic follow-up study, 1971-1992. National Health and Nutrition Examination Survey. *Jama* 2000;283:2404-10.
5. Keenan T, Blaha MJ, Nasir K, et al. Relation of uric acid to serum levels of high-sensitivity C-reactive protein, triglycerides, and high-density lipoprotein cholesterol and to hepatic steatosis. *Am J Cardiol* 2012;110:1787-92.
6. See LC, Kuo CF, Chuang FH, et al. Hyperuricemia and metabolic syndrome: associations with chronic kidney disease. *Clin Rheumatol* 2011;30:323-30.
7. Sirota JC, McFann K, Targher G, et al. Elevated serum uric acid levels are associated with non-alcoholic fatty liver disease independently of metabolic syndrome features in the United States: Liver ultrasound data from the National Health and Nutrition Examination Survey. *Metabolism* 2013;62:392-9.
8. Gamala M, Jacobs JWG. Gout and hyperuricaemia: a worldwide health issue of joints and beyond. *Rheumatology (Oxford)* 2019;58:2083-85.
9. Han B, Wang N, Chen Y, et al. Prevalence of hyperuricaemia in an Eastern Chinese population: a cross-sectional study. *BMJ Open* 2020;10:e035614.
10. Huang XB, Zhang WQ, Tang WW, et al. Prevalence and associated factors of hyperuricemia among urban adults aged 35-79 years in southwestern China: a community-based cross-sectional study. *Sci Rep* 2020;10:15683.
11. Gee Teng G, Pan A, Yuan JM, et al. Cigarette Smoking and the Risk of Incident Gout in a Prospective Cohort Study. *Arthritis Care Res (Hoboken)* 2016;68:1135-42.
12. Han QX, Zhang D, Zhao YL, et al. Risk Factors For Hyperuricemia In Chinese Centenarians And Near-Centenarians. *Clin Interv Aging* 2019;14:2239-47.
13. Hirotsu C, Tufik S, Guindalini C, et al. Association between uric acid levels and obstructive sleep apnea syndrome in a large epidemiological sample. *PLoS One* 2013;8:e66891.
14. Shi T, Min M, Sun C, et al. A meta-analysis of the association between gout, serum uric acid level, and obstructive sleep apnea. *Sleep Breath* 2019;23:1047-57.
15. Wiener RC, Shankar A. Association between Serum Uric Acid Levels and Sleep Variables: Results from the National Health and Nutrition Survey 2005-2008. *Int J Inflam* 2012;2012:363054.
16. Xiong X, He F, Sun G, et al. The relationship between self-reported habitual snoring and hyperuricemia among Chinese urban adults: a cross-sectional study. *Sleep Med* 2020;68:207-12.

For peer review only - http://bmjopen.bmj.com/site/about/guidelines.xhtml

17. Zheng C, Song H, Wang S, et al. Serum Uric Acid Is Independently Associated with Risk of Obstructive Sleep Apnea-Hypopnea Syndrome in Chinese Patients with Type 2 Diabetes. *Dis Markers* 2019;2019:4578327.
18. Zhao X, Hong F, Yin J, et al. Cohort profile: the China Multi-Ethnic cohort (CMEC) study. *Int J Epidemiol* 2020
19. Cai Z, Xu X, Wu X, et al. Hyperuricemia and the metabolic syndrome in Hangzhou. *Asia Pac J Clin Nutr* 2009;18:81-7.
20. Fang S, Zhou J. Association of Daytime Napping and Diagnosed Diabetes in Middle-Aged Premenopausal, Middle-Aged Postmenopausal, and Older Postmenopausal Chinese Women. *Am J Health Promot* 2019;33:1107-14.
21. Xiao M, Tang X, Zhang F, et al. Association between self-reported snoring and hypertension among Chinese Han population aged 30-79 in Chongqing, China. *Environ Health Prev Med* 2020;25:78.
22. Swartz AM, Strath SJ, Bassett DR, Jr., et al. Estimation of energy expenditure using CSA accelerometers at hip and wrist sites. *Med Sci Sports Exerc* 2000;32:S450-6.
23. Chinese Joint Committee on the Revision of Guidelines for Prevention and Treatment of Adult Dyslipidemia. guidelines for prevention and treatment of dyslipidemia in Chinese adults（2016 vison）. *Chin Circ J* 2016;31:937-53.
24. 2. Classification and Diagnosis of Diabetes. *Diabetes Care* 2017;40:S11-s24.
25. Chobanian AV, Bakris GL, Black HR, et al. The Seventh Report of the Joint National Committee on Prevention, Detection, Evaluation, and Treatment of High Blood Pressure: the JNC 7 report. *Jama* 2003;289:2560-72.
26. Harrison R. Structure and function of xanthine oxidoreductase: where are we now? *Free Radic Biol Med* 2002;33:774-97.
27. Gozal D, Kheirandish L. Oxidant stress and inflammation in the snoring child: confluent pathways to upper airway pathogenesis and end-organ morbidity. *Sleep Med Rev* 2006;10:83-96.
28. Puig F, Rico F, Almendros I, et al. Vibration enhances interleukin-8 release in a cell model of snoring-induced airway inflammation. *Sleep* 2005;28:1312-6.
29. Real JT, Martínez-Hervás S, García-García AB, et al. Circulating mononuclear cells nuclear factor-kappa B activity, plasma xanthine oxidase, and low grade inflammatory markers in adult patients with familial hypercholesterolaemia. *Eur J Clin Invest* 2010;40:89-94.
30. Zanetti M, Zenti M, Barazzoni R, et al. HELP LDL apheresis reduces plasma pentraxin 3 in familial hypercholesterolemia. *PLoS One* 2014;9:e101290.
31. Ma J, Zhang H, Wang H, et al. Association Between Self-Reported Snoring and Metabolic Syndrome: A Systematic Review and Meta-Analysis. *Front Neurol* 2020;11:517120.
32. Zhang N, Chen Y, Chen S, et al. Self-Reported Snoring Is Associated with Dyslipidemia, High Total Cholesterol, and High Low-Density Lipoprotein Cholesterol in Obesity: A Cross-Sectional Study from a Rural Area of China. *Int J Environ Res Public Health* 2017;14
33. Dai N, Shi Q, Hua Y, et al. Snoring frequency and risk of type 2 diabetes mellitus: a prospective cohort study. *BMJ Open* 2021;11:e042469.
34. Lee SK, Choi K, Chang YH, et al. Increased risk for new-onset hypertension in midlife male snorers: The 14-year follow-up study. *J Sleep Res* 2019;28:e12757.
35. Qi D, Liu J, Wang C, et al. Sex-specific differences in the prevalence of and risk factors for

For peer review only - http://bmjopen.bmj.com/site/about/guidelines.xhtml

hyperuricemia among a low-income population in China: a cross-sectional study. *Postgrad Med* 2020;132:559-67.

36. Chen Y, Xia Y, Han X, et al. Association between serum uric acid and atrial fibrillation: a cross-sectional community-based study in China. *BMJ Open* 2017;7:e019037.
37. Qiu L, Cheng XQ, Wu J, et al. Prevalence of hyperuricemia and its related risk factors in healthy adults from Northern and Northeastern Chinese provinces. *BMC Public Health* 2013;13:664.
38. Liu H, Zhang XM, Wang YL, et al. Prevalence of hyperuricemia among Chinese adults: a national cross-sectional survey using multistage, stratified sampling. *J Nephrol* 2014;27:653-8.
39. Jennum P, Hein HO, Suadicani P, et al. Cardiovascular risk factors in snorers. A cross-sectional study of 3,323 men aged 54 to 74 years: the Copenhagen Male Study. *Chest* 1992;102:1371-6.

For peer review only

Figure 1 Data cleaning flow chart

For peer review only - http://bmjopen.bmj.com/site/about/guidelines.xhtml

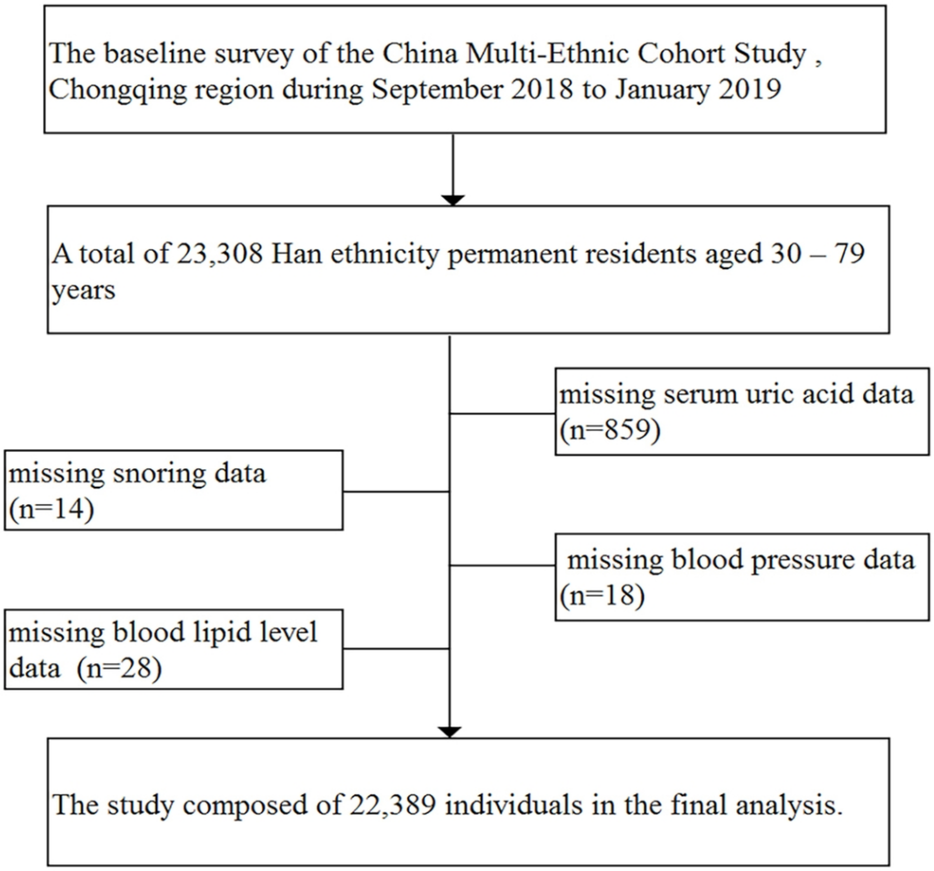

Figure 1 Data cleaning flow chart

101x101mm (300 x 300 DPI)

STROBE Statement—Checklist of items that should be included in reports of ***cross-sectional studies***

	Item No	Recommendation	Page No
**Title and abstract**	1	(*a*) Indicate the study's design with a commonly used term in the title or the abstract	1
		(*b*) Provide in the abstract an informative and balanced summary of what was done and what was found	2-3
**Introduction**			
Background/rationale	2	Explain the scientific background and rationale for the investigation being reported	3-4
Objectives	3	State specific objectives, including any prespecified hypotheses	4
**Methods**			
Study design	4	Present key elements of study design early in the paper	4
Setting	5	Describe the setting, locations, and relevant dates, including periods of recruitment, exposure, follow-up, and data collection	4-5
Participants	6	(*a*) Give the eligibility criteria, and the sources and methods of selection of participants	5
Variables	7	Clearly define all outcomes, exposures, predictors, potential confounders, and effect modifiers. Give diagnostic criteria, if applicable	5-7
Data sources/ measurement	8*	For each variable of interest, give sources of data and details of methods of assessment (measurement). Describe comparability of assessment methods if there is more than one group	5-6
Bias	9	Describe any efforts to address potential sources of bias	5
Study size	10	Explain how the study size was arrived at	5
Quantitative variables	11	Explain how quantitative variables were handled in the analyses. If applicable, describe which groupings were chosen and why	5-7
Statistical methods	12	(*a*) Describe all statistical methods, including those used to control for confounding	7-8，12
		(*b*) Describe any methods used to examine subgroups and interactions	8
		(*c*) Explain how missing data were addressed	5
		(*d*) If applicable, describe analytical methods taking account of sampling strategy	7-8
		(*e*) Describe any sensitivity analyses	
**Results**			
Participants	13*	(a) Report numbers of individuals at each stage of study—eg numbers potentially eligible, examined for eligibility, confirmed eligible, included in the study, completing follow-up, and analysed	5
		(b) Give reasons for non-participation at each stage	5
		(c) Consider use of a flow diagram	5
Descriptive data	14*	(a) Give characteristics of study participants (eg demographic, clinical, social) and information on exposures and potential confounders	8-10
		(b) Indicate number of participants with missing data for each variable of interest	
Outcome data	15*	Report numbers of outcome events or summary measures	8
Main results	16	(*a*) Give unadjusted estimates and, if applicable, confounder-adjusted estimates and their precision (eg, 95% confidence interval). Make clear which confounders were adjusted for and why they were included	11-12

For peer review only - http://bmjopen.bmj.com/site/about/guidelines.xhtml
For peer review only

		(*b*) Report category boundaries when continuous variables were categorized	5-7
		(*c*) If relevant, consider translating estimates of relative risk into absolute risk for a meaningful time period	11-12
Other analyses	17	Report other analyses done—eg analyses of subgroups and interactions, and sensitivity analyses	11-12
**Discussion**			
Key results	18	Summarise key results with reference to study objectives	13
Limitations	19	Discuss limitations of the study, taking into account sources of potential bias or imprecision. Discuss both direction and magnitude of any potential bias	16
Interpretation	20	Give a cautious overall interpretation of results considering objectives, limitations, multiplicity of analyses, results from similar studies, and other relevant evidence	13-16
Generalisability	21	Discuss the generalisability (external validity) of the study results	16
**Other information**			
Funding	22	Give the source of funding and the role of the funders for the present study and, if applicable, for the original study on which the present article is based	17

*Give information separately for exposed and unexposed groups.

Note: An Explanation and Elaboration article discusses each checklist item and gives methodological background and published examples of transparent reporting. The STROBE checklist is best used in conjunction with this article (freely available on the Web sites of PLoS Medicine at http://www.plosmedicine.org/, Annals of Internal Medicine at http://www.annals.org/, and Epidemiology at http://www.epidem.com/). Information on the STROBE Initiative is available at www.strobe-statement.org.

For peer review only

For peer review only - http://bmjopen.bmj.com/site/about/guidelines.xhtml

BMJ Open

Association of self-reported snoring and hyperuricemia: a large cross-sectional study in Chongqing, China

Journal:	*BMJ Open*
Manuscript ID	bmjopen-2021-056143.R1
Article Type:	Original research
Date Submitted by the Author:	25-Dec-2021
Complete List of Authors:	chen, ting; Chongqing Medical University; Chongqing Municipal Center for Disease Control and Prevention<br>Ding, Xianbin; Chongqing Municipal Center for Disease Control and Prevention<br>Tang, Wenge; Chongqing Municipal Center for Disease Control and Prevention<br>Chen, Liling; Chongqing Municipal Center for Disease Control and Prevention<br>Mao, Deqiang; Chongqing Municipal Center for Disease Control and Prevention<br>Song, Lingling; Chongqing Medical University, Department of Nutrition and Food Hygiene<br>Lian, Xuemei; Chongqing Medical University, Department of Nutrition and Food Hygiene
**Primary Subject Heading**:	Public health
Secondary Subject Heading:	Epidemiology
Keywords:	EPIDEMIOLOGY, PREVENTIVE MEDICINE, PUBLIC HEALTH, Other metabolic, e.g. iron, porphyria < DIABETES & ENDOCRINOLOGY

SCHOLARONE™
Manuscripts

I, the Submitting Author has the right to grant and does grant on behalf of all authors of the Work (as defined in the below author licence), an exclusive licence and/or a non-exclusive licence for contributions from authors who are: i) UK Crown employees; ii) where BMJ has agreed a CC-BY licence shall apply, and/or iii) in accordance with the terms applicable for US Federal Government officers or employees acting as part of their official duties; on a worldwide, perpetual, irrevocable, royalty-free basis to BMJ Publishing Group Ltd ("BMJ") its licensees and where the relevant Journal is co-owned by BMJ to the co-owners of the Journal, to publish the Work in this journal and any other BMJ products and to exploit all rights, as set out in our licence.

The Submitting Author accepts and understands that any supply made under these terms is made by BMJ to the Submitting Author unless you are acting as an employee on behalf of your employer or a postgraduate student of an affiliated institution which is paying any applicable article publishing charge ("APC") for Open Access articles. Where the Submitting Author wishes to make the Work available on an Open Access basis (and intends to pay the relevant APC), the terms of reuse of such Open Access shall be governed by a Creative Commons licence – details of these licences and which Creative Commons licence will apply to this Work are set out in our licence referred to above.

Other than as permitted in any relevant BMJ Author's Self Archiving Policies, I confirm this Work has not been accepted for publication elsewhere, is not being considered for publication elsewhere and does not duplicate material already published. I confirm all authors consent to publication of this Work and authorise the granting of this licence.

For peer review only

Title: Association of self-reported snoring and hyperuricemia: a large cross-sectional study in Chongqing, China

Authors: Ting Chen[1,2], Xianbin Ding[2] ,Wenge Tang[2], Liling Chen[2], Deqiang Mao[2], Lingling Song[1] ,Xuemei Lian[1*]

Affiliations:

1 Department of Nutrition and Food Hygiene, School of Public Health and Management, Chongqing Medical University, Chongqing, China

2 Chongqing Center for Disease Control and Prevention, Chongqing, China

*** Corresponding authors:**

Xuemei Lian, No.1 Yixueyuan Rd, Yuzhong District, Chongqing 400016, China, Email: xuemeilian@cqmu.edu.cn

For peer review only

For peer review only - http://bmjopen.bmj.com/site/about/guidelines.xhtml

Abstract

Objective: To examine the relationship between self-reported snoring and hyperuricemia in a large-scale population in Chongqing, China.

Setting: Face-to-face electronic questionnaire survey, physical examination and biological sample testing were conducted in 13 districts of Chongqing. Chongqing is a municipality in southwest China.

Participants: In this study, 23,308 Han ethnicity permanent residents aged 30 – 79 years were recruited. Individuals missing data were excluded, 22,389 subjects were included in final analysis.

Primary and secondary outcome measures: Serum uric acid was measured using an oxidase method. Hyperuricemia was defined as serum UA >420 µmol/L in men and >360 µmol/L in women. Information about self-reported snoring was obtained by questionnaire survey. All participants were divided into "no snoring" "snoring occasionally" and "snoring frequently". Multivariable logistic regression analysis was performed to assess the relationship between self-reported snoring and hyperuricemia.

Results: The prevalence of hyperuricemia was 14.43%, and snorers were more likely to have hyperuricemia than non-snorer in different age and gender groups. For the total population, those who snore occasionally or frequently were more likely to be hyperuricemia (OR 1.17, 95% CI 1.05-1.29; OR 1.32, 95% CI 1.19-1.46) compared with no snoring people. Stratification by age, gender and BMI, we found that the positive association between snoring frequently and hyperuricemia was insisted in different age, gender and high BMI groups, and the strength of association varied with

For peer review only - http://bmjopen.bmj.com/site/about/guidelines.xhtml

different age, gender and BMI category.

Conclusion: Snoring frequency was positively associated with higher risk of hyperuricemia. People who snore frequently should pay close attention to uric acid level in order to achieve early prevention of gout, particularly for snorers who are female, aged over 59 and overweight or obesity.

Keywords: Self-reported snoring, Hyperuricemia, Chinese Han population

Strengths and limitations of this study:

The sample size of the study was relatively large.

This study analysis was thorough and comprehensive.

The cross-sectional data analysis limited ability to explore the casual relationship.

Our study results were limited by age and ethnicity.

Snoring frequency data was subjective.

Introduction

Hyperuricemia is a common chronic disease caused by purines metabolism dysfunction and uric acid excretion disorder. Hyperuricemia is becoming an important public health issue with its increasing prevalence in China. A meta-analysis study including 177 eligible studies between 2000 and 2019 reported that the prevalence of hyperuricemia was 16.4% in mainland China [1]. Previous studies have confirmed that hyperuricemia not only is associated with the development of gout, but also increases risks of vascular diseases, diabetes mellitus, kidney disease, and metabolic syndrome [2-7]. Given the large disease burden related to hyperuricemia [8], it is necessary to recognize more risk factors correlated with it.

Meanwhile, various major risk factors for hyperuricemia have been found, such as

For peer review only - http://bmjopen.bmj.com/site/about/guidelines.xhtml

low economic status [9], obesity [10], and smoking [11], and some studies have found a link between sleep-disordered breathing and hyperuricemia [12-17]. However, the association of snoring and hyperuricemia has been studied much less extensively. To our knowledge, only three studies [12 15 16] have been referred to explore the relationship of snoring and hyperuricemia. In addition, the three studies had small sample sizes, considered confounding factors insufficiently and did not examine the association by age or BMI category stratified analysis.

This study aimed to examine comprehensively the relationship of self-reported snoring and hyperuricemia after controlling for potential confounding factors in a large-scale population in Chongqing, China.

Methods

Participants

The present study was from the baseline survey of the China Multi-Ethnic Cohort Study [18], Chongqing region, which is a collaborated study by Sichuan University, Chongqing Medical University and Chongqing Center for Disease Control and Prevention. This study was carried out in 13 districts of Chongqing, including Yuzhong District, Jiulongpo District, Nan·an District, Ba·nan District, Changshou District, Jiangjin District, Hechuan District, Qijiang District, Dazu District, Tongnan District, Rongchang District, Wulong District, Fengdu District. In brief, a face-to-face electronic questionnaire survey, physical examination and biological sample testing were conducted in this study. In order to ensure the quality of research, strict quality control measures were applied, for example checking electronic recording. A total of

For peer review only - http://bmjopen.bmj.com/site/about/guidelines.xhtml

23,308 Han ethnicity permanent residents aged 30 – 79 years were recruited by using randomized sampling during September 2018 to January 2019. The sample inclusion criteria and exclusion criteria have been described in detail previously [18]. We excluded individual data missing serum uric acid (n=859) or snoring (n=14) or blood pressure (n=18) or blood lipid level (n=28). Finally, this present study consisted of 22,389 (figure 1) individuals providing complete information. All participants had written informed consent prior to the study. This study was approved by the ethics committee of Sichuan University (No.K2016038).

Measures

Serum uric acid (UA) measurement and the definition of hyperuricemia

After fasting for 12 hours, venous blood samples were collected from all participants. Within 2 hours after collection, blood samples were transported in dry ice to one medical laboratory certified by Chongqing Center for Disease Control and Prevention. Serum UA was measured with a Beckman Coulter AU 680 device using an oxidase method in Chongqing Di·an Medical Laboratory Center. The validity of serum UA assessment was described by relative deviation, which was controlled within 10%. The coefficient of variations of serum UA assessment was ⩽ 4.0% within the group. Hyperuricemia was defined as serum UA >420 μmol/L in men and >360 μmol/L in women [9 19].

Measurement of snoring

Information about self-reported snoring was obtained by the question “Do you snore when you have a sleep?” Then, the frequency of snoring (“occasionally” and

For peer review only - http://bmjopen.bmj.com/site/about/guidelines.xhtml

“frequently”) was asked for those who answered “yes”. Finally, all participants were divided into “no snoring” “snoring occasionally” and “snoring frequently”.

Assessment of covariates

The present study included sociodemographic characteristics such as age, gender (male, female), marital status, education level, residential region (urban, rural), employment status (yes, no), household yearly income level (＜60,000 RMB,⩾ 60,000 RMB). Consistent with previous studies in China [20 21], participates were grouped into low-aged adults (＜45 years), middle-aged adults (45-59 years) and older adults (＞59 years). Marital status was categorized into married or living with partner and others, including widowed, unmarried, divorced or separated. Education level was classified into primary or below (elementary school or illiterate), secondary (junior school, senior school or secondary vocational school) and college or above.

Moreover, the present study collected information about health-related behaviours. Smoking status was grouped into never smoker, current smoker and ever smoker. Alcohol drinking status was categorized into never or hardly drinker, occasionally drinker (only drink on special occasions or the frequency less than once a week), often drinker (at least once a week in the past year). Tea and sugar-sweetened beverage drinking status was defined by the question “Have you ever been drinking tea/ sugar-sweetened beverage weekly for more than half a year?” Accordingly, participants were divided into drinker and non-drinker. Oil and salt intake were collected by asking about the consumption of oil and salt per month in the past year. Energy intake was measured using a semi-quantitative standardized food frequency

For peer review only - http://bmjopen.bmj.com/site/about/guidelines.xhtml

questionnaire with 13 food categories, including red meat, aquatic/sea food products and so on. Red meat intake was created as a dichotomized variable with 350 g/week as the cut-point according to median. Similarly aquatic/sea food products intake was also grouped into "≤93.33 g/week" and ">93.33 g/week". Physical activity level (expressed as metabolic equivalent, MET) was estimated based on self-reported activities using a Compendium of Physical Activities [22], and the physical activities questionnaire referenced the global physical activity questionnaire. Nocturnal sleep and midday nap duration were collected according to the two questions: "how many hours do you usually nocturnal sleep every day in the past month ?" and "how many minutes do you usually midday nap every day in the past month ?"

Lastly, the information regarding clinic variables were obtained including body mass index (BMI), kidney function, dyslipidaemia, diabetes and hypertension. BMI was calculated as weight divided by height^2 (kg/m^2) and grouped into "<24" "24 – 27.9" and "≥28" [16]. Kidney function was stratified 5 stages by estimated glomerular filtration (eGFR): Chronic kidney disease 1(CKD 1) (eGFR >90 ml/min), CKD 2 (eGFR 60–89 ml/min), CKD 3(eGFR 30–59 ml/min), CKD 4 (eGFR 15–29 ml/min), CKD 5 (eGFR <15 ml/min) and eGFR was estimated by serum creatinine with the abbreviated MDRD formula (186 × (serum creatinine/88.4)$^{-1.154}$ × (age)$^{-0.203}$ × (0.742 if female)) [23]. Dyslipidaemia was defined using serum total cholesterol concentration of ≥6.2mmol/l, triacylglycerol concentration of ≥2.3 mmol/l, low-density lipoprotein cholesterol concentration of ≥4.1 mmol/l, high-density lipoprotein cholesterol concentration of < 1.0 mmol/l or a self-reported diagnosis of

For peer review only - http://bmjopen.bmj.com/site/about/guidelines.xhtml

dyslipidaemia[24]. Diabetes was diagnosed as fasting blood glucose level of ⩾7.0 mmol/l, glycosylated haemoglobin percentage of ⩾6.5% or a self-reported diagnosis of diabetes[25]. Participants with were regarded as having hypertension if any of the following three items were met: systolic blood pressure of ⩾140 mmHg, diastolic blood pressure of ⩾ 90 mmHg or self-reported diagnosis of hypertension[26].

Statistical analysis

The analyses were performed with the SPSS, version 25.0. Characteristics of the participants, including sociodemographic characteristics, health-related behaviours and clinic variables, were compared according to the frequency of snoring ("no", "occasionally" and "frequently"). All continuous data were described as mean± standard deviations, while categorical data were summarized as percentages. The one-way analysis of variance was used to compare the difference in continuous data, and chi-square test was used to compare the difference in categorical data. Then, the prevalence of hyperuricemia with different snoring frequency was examined using the chi-square test in different age and gender groups. Finally, Multivariable logistic regression analysis was performed to evaluate the relationship between snoring and hyperuricemia after adjusting for different covariates in different age groups, gender groups , BMI groups and total population, and odds ratio (OR) and 95% confidence interval (CI) for the outcome variable were calculated. Two-sided *p value of* <0.05 was considered to be statistically significant.

Patient and public involvement

Patients and the public were not involved in the design of the study, recruitment and

For peer review only - http://bmjopen.bmj.com/site/about/guidelines.xhtml

conduct of the study or dissemination of the study results.

Results

Sample characteristics of participants

The characteristics of participants based on the categories of self-reported snoring are showed in Table 1.The study composed of 22,389 individuals in the final analysis. Of the study subjects, 51.23%, 26.71%, 22.06% reported "no snoring", "snoring occasionally", "snoring frequently", respectively. Participants who snore were more likely to be aged 45–59, male, employed person, non-smokers, occasionally alcohol drinkers, non-tea drinkers. In addition, compared with those who reported "no snoring", snorers had a higher level of energy take, midday nap duration, dyslipidaemia, diabetes, hypertension, but had a lower level of physical activity, nocturnal sleep duration.

Table 1 Characteristics of participants based on the categories of self-reported snoring

Characteristics	Self-reported snoring			*P* value
	No	Occasionally	Frequently	
Number of subjects	11469	5981	4939	
**Sociodemographic Characteristics**				
Age				< 0.001
< 45	36.18	30.16	19.80	
45-59	39.64	42.37	43.59	
> 59	24.18	27.47	36.61	
Male	35.26	53.02	66.13	< 0.001
Married or living with partner	87.75	88.13	88.01	0.742

For peer review only - http://bmjopen.bmj.com/site/about/guidelines.xhtml

Education level				< 0.001
Primary or below	33.16	28.57	36.83	
Secondary	49.94	53.10	49.20	
College or above	16.90	18.32	13.97	
Urban	27.49	33.42	25.81	< 0.001
Employment	63.05	62.43	59.73	
Income (< 60,000 RMB)	59.51	55.12	59.91	< 0.001
**Health-related behaviours**				
Smoking				< 0.001
Never	80.99	69.27	59.71	
Current	14.61	23.61	31.00	
Ever	4.40	7.12	9.29	
Alcohol drinking				< 0.001
Never or hardly	53.00	38.40	37.84	
Occasionally	37.56	45.23	40.27	
Often	9.44	16.37	21.89	
Tea drinker	16.27	24.33	28.10	< 0.001
Sugar-sweetened beverage drinker	1.96	2.51	3.52	< 0.001
Oil intake (g/week)	56.71±0.30	56.66±0.42	58.33+0.47	0.008
Salt intake (g/week)	48.36±0.27	47.45±0.36	51.04±0.42	< 0.001
Energy intake (kcal/week)	12776.57±39.74	13241.66±56.36	13838.48±64.34	< 0.001
red meat intake (g/week）				< 0.001
≤350	53.38	50.83	44.97	
＞350	46.62	49.17	55.03	
Aquatic/sea food products intake (g/week）				< 0.001
≤ 93.33	52.11	47.82	47.60	
> 93.33	47.89	52.18	52.40	
Physical activity (MET minutes /week)	10176.77±63.98	9731.86±86.57	9777.99±101.00	< 0.001

For peer review only

Nocturnal sleep duration (h/day)	7.18±0.01	7.04±0.02	6.97±0.02	< 0.001
Midday nap duration (min/day)	28.91±0.33	31.17±0.45	33.43±0.54	< 0.001
**Clinic variables**				
BMI ((kg/m^2))				< 0.001
＜24	55.07	39.26	24.20	
24-27.9	35.91	45.81	47.99	
⩾28	9.02	14.93	27.82	
Kidney function				
CKD1	71.94	68.02	64.08	< 0.001
CKD2	27.07	30.80	34.06	
CKD3	0.95	1.10	1.70	
CKD4	0.02	0.05	0.12	
CKD5	0.02	0.03	0.04	
Dyslipidaemia	23.99	33.46	44.26	< 0.001
Diabetes	7.35	10.18	15.02	< 0.001
Hypertension	28.13	36.23	50.50	< 0.001

Continuous data were described as mean± standard deviations, and statistical significance was assessed by the one-way analysis of variance.

Categorical data were summarized as percentages (%), and statistical significance was assessed by chi-square test.

The prevalence of hyperuricemia

The prevalence of hyperuricemia according to different snoring frequency with different groups is presented in Table 2. In all, the prevalence of hyperuricemia was 14.43% (3230/22389) in our study population. Compared with the “no snoring” group, the prevalence of hyperuricemia was higher in the “snoring occasionally” and “snoring frequently” groups among the three age groups (P<0.05). In addition, those who reported “snoring occasionally” or “snoring frequently” were more likely to have hyperuricemia than “no snoring” group in different gender groups (P<0.05). Similarly,

For peer review only

For peer review only - http://bmjopen.bmj.com/site/about/guidelines.xhtml

in the total population, higher prevalence of hyperuricemia was found in the "snoring occasionally" or "snoring frequently" compared with "no snoring" group.

Table 2 Prevalence of hyperuricemia according to snoring frequency in different groups

Snoring frequency	Age [n (%)]			Gender [n (%)]		Total
	< 45	45-59	> 59	Male	Female	
No	376(9.06)	441(9.70)	358(12.91)	650(16.07)	525(7.07)	1175(10.25)
Occasionally	326(18.07)	370(14.60)	261(15.89)	659(20.78)	298(10.60)	957(16.00)
Frequently	299(30.57)	452(20.99)	347(19.19)	820(25.11)	278(16.62)	1098(22.23)
χ2	322.34	160.32	33.23	91.72	156.38	418.14
P value	< 0.001	< 0.001	< 0.001	< 0.001	< 0.001	< 0.001

Association self-reported snoring and hyperuricemia

Results of multivariable logistic regression analysis about the association self-reported snoring and hyperuricemia are showed in Table 3. In the total population, after adjusting for sociodemographic characteristics, health-related behaviours and clinic variables, those who reported "snoring occasionally" or "snoring frequently" were more likely to be hyperuricemia (OR 1.17, 95%CI 1.05-1.29; OR 1.32, 95%CI 1.19-1.46, respectively) compared with "no snoring" group. The stratified analysis by age results showed: after adjusting for all variables, in three age groups higher odd ratio of hyperuricemia (OR 1.29, 95%CI 1.04-1.59; OR 1.24, 95%CI 1.05-1.46; OR 1.34, 95%CI 1.12-1.59, respectively) was found in the "snoring frequently" group compared with "no snoring" group. The stratified analysis by gender results showed: after adjusting for all variables, those who reported "snoring frequently" had a higher odd ratio of hyperuricemia in male (OR 1.23, 95%CI 1.08-1.40) and female (OR 1.35,

For peer review only - http://bmjopen.bmj.com/site/about/guidelines.xhtml

95%CI 1.13-1.62, respectively) than “no snoring” group. The stratified analysis by BMI results showed: after adjusting for all variables, those who reported “snoring frequently” remained at an increased risk of hyperuricemia in BMI 24-27.9 and ⩾28 kg/m^2 groups (OR 1.33, 95%CI 1.15-1.55; OR 1.38, 95%CI 1.13-1.69), but the association was not found in BMI ＜24 kg/m^2 group (P>0.05).

Table 3 Association self-reported snoring and hyperuricemia

Variables	NO	Occasionally		Frequently	
	OR（95%CI）	OR（95%CI）	*P* value	OR（95%CI）	*P* value
**Age**					
**< 45**					
Model 1	1.00(reference)	2.21(1.89,2.60)	< 0.001	4.42(3.72,5.25)	< 0.001
Model 2	1.00(reference)	1.51(1.28,1.79)	< 0.001	2.44(2.03,2.94)	< 0.001
Model 3	1.00(reference)	1.48(1.25,1.76)	< 0.001	2.39(1.97,2.88)	< 0.001
Model 4	1.00(reference)	1.14(0.94,1.37)	0.18	**1.29(1.04,1.59)**	**0.02**
**45-59**					
Model 1	1.00(reference)	1.59(1.37,1.85)	< 0.001	2.47(2.14,2.85)	< 0.001
Model 2	1.00(reference)	1.33(1.14,1.55)	< 0.001	1.88(1.62,2.18)	< 0.001
Model 3	1.00(reference)	1.29(1.11,1.50)	< 0.001	1.83(1.58,2.13)	< 0.001
Model 4	1.00(reference)	1.07(0.91,1.26)	0.40	**1.24(1.05,1.46)**	**0.01**
**> 59**					
Model 1	1.00(reference)	1.28(1.07,1.52)	0.01	1.60(1.36,1.88)	< 0.001
Model 2	1.00(reference)	1.25(1.05,1.48)	0.01	1.59(1.35,1.87)	< 0.001
Model 3	1.00(reference)	1.22(1.03,1.46)	0.02	1.59(1.34,1.87)	< 0.001
Model 4	1.00(reference)	1.18(0.98,1.42)	0.08	**1.34(1.12,1.59)**	**0.00**

For peer review only

For peer review only - http://bmjopen.bmj.com/site/about/guidelines.xhtml

**Gender**					
**Male**					
Model 1	1.00(reference)	1.37(1.22,1.54)	< 0.001	1.75(1.56,1.96)	< 0.001
Model 2	1.00(reference)	1.30(1.15,1.47)	< 0.001	1.79(1.59,2.00)	< 0.001
Model 3	1.00(reference)	1.25(1.11,1.41)	< 0.001	1.72(1.53,1.93)	< 0.001
Model 4	1.00(reference)	1.07(0.94,1.22)	0.32	**1.23(1.08,1.40)**	**0.00**
**Female**					
Model 1	1.00(reference)	1.56(1.34,1.81)	< 0.001	2.62(2.24,3.06)	< 0.001
Model 2	1.00(reference)	1.42(1.22,1.65)	< 0.001	2.13(1.81,2.50)	< 0.001
Model 3	1.00(reference)	1.42(1.22,1.66)	< 0.001	2.13(1.81,2.50)	< 0.001
Model 4	1.00(reference)	1.18(1.00,1.39)	0.05	**1.35(1.13,1.62)**	**0.00**
**BMI**					
**＜24**					
Model 1	1.00(reference)	1.48(1.25,1.76)	< 0.001	1.96(1.61,2.40)	< 0.001
Model 2	1.00(reference)	1.22(1.02,1.45)	0.03	1.39(1.12,1.71)	0.00
Model 3	1.00(reference)	1.20(1.00,1.43)	0.05	1.37(1.11,1.69)	0.00
Model 4	1.00(reference)	1.13(0.94,1.35)	0.20	1.22(0.98,1.52)	0.08
**24-27.9**					
Model 1	1.00(reference)	1.43(1.25,1.64)	< 0.001	1.84(1.61,2.10)	< 0.001
Model 2	1.00(reference)	1.21(1.05,1.39)	0.01	1.50(1.30,1.73)	< 0.001
Model 3	1.00(reference)	1.19(1.03,1.37)	0.02	1.48(1.28,1.71)	< 0.001
Model 4	1.00(reference)	1.12(0.97,1.30)	0.12	**1.33(1.15,1.55)**	**< 0.001**
**⩾28**					
Model 1	1.00(reference)	1.42(1.16,1.74)	0.00	1.68(1.40,2.02)	< 0.001
Model 2	1.00(reference)	1.30(1.06,1.61)	0.01	1.47(1.22,1.78)	< 0.001
Model 3	1.00(reference)	1.29(1.04,1.59)	0.02	1.48(1.22,1.79)	< 0.001
Model 4	1.00(reference)	1.22(0.98,1.52)	0.08	**1.38(1.13,1.69)**	**0.00**

For peer review only

For peer review only - http://bmjopen.bmj.com/site/about/guidelines.xhtml

**Total**					
Model 1	1.00(reference)	1.67(1.52,1.83)	< 0.001	2.50(2.29,2.74)	< 0.001
Model 2	1.00(reference)	1.45(1.32,1.59)	< 0.001	2.03(1.84,2.23)	< 0.001
Model 3	1.00(reference)	1.43(1.30,1.57)	< 0.001	2.00(1.82,2.20)	< 0.001
Model 4	1.00(reference)	**1.17(1.05,1.29)**	**0.00**	**1.32(1.19,1.46)**	**<0.001**

Model 1: unadjusted model
Model 2: adjusted for gender (when stratification by gender, it was included), age (when stratification by age, it was included), marital status, education level, residential region, employment status and household yearly income level
Model 3: additionally adjusted for smoking status, alcohol drinking status, tea drinking status, sugar-sweetened beverage drinking status, oil intake, salt intake, energy intake, red meat intake, aquatic/sea food products intake, physical activity level, nocturnal sleep and midday nap duration
Model 4: additionally BMI, kidney function, dyslipidaemia, diabetes and hypertension

Discussion

In our study, we found that self-reported snoring was independently associated with hyperuricemia in total population. After adjusting for the sociodemographic characteristics, health-related behaviours and clinic variables, those who reported “snoring occasionally” and “snoring frequently” had 17% and 32% increased risk of hyperuricemia compared with “no snoring” group, respectively.

Our findings are consistent with those results of previous studies. An analysis of 6491 participants from the National Health and Nutrition Survey 2005–2008 found an increased risk of hyperuricemia associated with snoring, which found similar results with our study[15]. Xiaolu et al. enrolled 7699 Chinese urban adults from Nanjing between September 2016 and February 2018, they verified the positive link between snoring and hyperuricemia [16]. A study of data from the baseline survey of the China Hainan Centenarian Cohort Study (CHCCS) also showed a positive association

For peer review only - http://bmjopen.bmj.com/site/about/guidelines.xhtml

between snoring and hyperuricemia in centenarians [12]. The possible mechanisms can partly explain the positive association between snoring and hyperuricemia. Snoring is often accompanied by oxygen desaturation leading to tissue hypoxia, and then this accelerates the breakdown of adenosine triphosphate, resulting in the accumulation of uric acid from purine catabolic product by xanthine dehydrogenase and xanthine oxidase [27]. In addition, some studies have showed that snoring is related to the inflammation response by vibration tissue cell releasing inflammatory factor [28 29], and previous study have found the association of inflammation response and metabolic disease, for example dyslipidaemia [30 31]. Therefore, whether the effect of snoring on hyperuricemia is mediated by inflammatory pathway needs to be further exploration.

Compared with previous studies, our study conducted more detailed relationship analysis of snoring and hyperuricemia in different age and gender groups. To our knowledge, no studies have been analysed age differences of association between snoring and hyperuricemia. We found that those who snore frequently with aged >59 year old had a 34% increased risk of hyperuricemia ($P < 0.05$) compared with no snoring people, and the strength of association was stronger than age 45- 59 (24%) and aged <45 (29%) groups, which might be related to decreasing metabolic function significantly in old people. This result suggested that people over 59 who snore frequently should pay more attention to their uric acid level in order to achieve early prevention of gout. In the present study, for male and female, those who snore frequently had 23% and 35% increased risk of hyperuricemia ($P < 0.05$) than no snoring people, respectively. Our findings showed the strength of association in

For peer review only - http://bmjopen.bmj.com/site/about/guidelines.xhtml

female was stronger compared with male, which are consistent with previous study [16]. To the best of our knowledge, no study has explored the possible mechanisms. It might be related to hormones difference in female and male. Therefore, more evidence studies on the mechanism of different strength relationship between snoring and hyperuricemia in female and male are needed. In this study, our results showed snoring occasionally and snoring frequently had increased risk of hyperuricemia than no snoring people in all different age and gender groups before adjusting for clinic variables, although the association between snoring occasionally and no snoring was not found for all groups after adjusting for all confounders. This implies that BMI, kidney function, dyslipidaemia, diabetes and hypertension may affect the relationship between snoring frequency and hyperuricemia in different age and gender groups. Previous studies had confirmed snoring to be significantly association with obesity [32], dyslipidaemia [33], diabetes [34] and hypertension [35]. In addition, those diseases may be related to serum uric acid level [36 37]. Therefore, it is necessary to adjust clinical indicators.

Besides, we conducted the stratified analysis by BMI. The result revealed that those who snore frequently with a high BMI remained at an increased risk of hyperuricemia compared with no snoring people, however, the positive association did not found in the low BMI group. The difference in results of three groups indicated the necessity to stratify by BMI. And this interesting result implies that overweight or obesity plays an important role in the relationship of snoring and hyperuricemia, which may be related to hypoxemia caused by overweight or obesity

For peer review only - http://bmjopen.bmj.com/site/about/guidelines.xhtml

[38].

In addition, our results showed that the prevalence of hyperuricemia was 14.43% in Chongqing, which was similar to the results of the Tianjin Brain Study (14.4%) [39]. However, this prevalence was more than that in the Tangshan population (13.4%) [40]. The difference may be explained by the different lifestyles and economic level in the two different areas, which are also closely related to hyperuricemia [41]. Our determined prevalence was also slightly higher than that in southwest (Chengdu and Chongqing) China hyperuricemia survey, which showed that the prevalence of hyperuricemia was 13.5% in 2013-2014[10]. This indicates that we should pay more attention to hyperuricemia due to the prevalence is increasing with the improvement of economic level and lifestyles changes.

Compared with previous study, our study is more thorough and comprehensive because the stratified analysis was conducted to explore the difference of relationship between snoring and hyperuricemia in different age, gender and BMI category groups. Only one previous study conducted a stratified analysis by gender, but this study did not consider to stratified analysis by age and BMI category [16]. Moreover, participants of the prior study were recruited from hospital, so the sample populations were not representative. Relatively, community-based large samples of our study increased the representativeness and generalizability of our result to some extent. To the best of our knowledge, this is the first study to explore the association between snoring and hyperuricemia used a large sample size in southwest China. Furthermore, the present study included many potential confounders, such as smoking, alcohol drinking, tea

For peer review only - http://bmjopen.bmj.com/site/about/guidelines.xhtml

drinking, sugar-sweetened beverage drinking, oil intake, salt intake, energy intake, red meat intake, aquatic/sea food products intake, physical activity, sleep duration, nap duration, BMI, kidney function, dyslipidaemia, diabetes and hypertension, which could minimize the bias.

The present study also has some limitations. First, our study data was a cross-sectional result from the cohort study, and the cross-sectional data analysis limited ability to explore the casual relationship between snoring and hyperuricemia. Further follow-up data analysis could confirm whether there was causal relationship between snoring and hyperuricemia by using special statistical model. Second, snoring frequency was obtained from a subjective self-reported question, which may be different with actual snoring frequency. However, Jennum et al. had found the self-reported snoring frequency was highly significant related to snoring record by microphone objectively [42], suggesting that the self-reported snoring could be applied in scientific research. In addition, self-reported snoring manner has also been used to other similar studies of the relationship between snoring and uric acid [12 15]. Third, the present study was lack of degree of snoring, which may distort the relationship between snoring and hyperuricemia. Thus, the degree of snoring should be considered in future studies. Fourth， our study was limited by age and ethnicity, so extrapolating the results to other age groups and ethnicity population should be noted.

Conclusions

In summary, snoring frequency was positively associated with higher risk of hyperuricemia in total population. In the three age groups, those who snore frequently

For peer review only - http://bmjopen.bmj.com/site/about/guidelines.xhtml

had increased risk of hyperuricemia compared with no snoring people, and the strength of association was the strongest in age >59 group. In addition, snore frequently is independently risk factor of hyperuricemia for male and female, and the strength of association in female was stronger compared with male. In high BMI population, those who snore frequently had increased risk of hyperuricemia compared with no snoring people, but the association was not found in BMI <24 groups. Our result indicated that people who snore frequently should pay close attention to uric acid level in order to achieve early prevention of gout, particularly for snorers who are female, aged over 59 and overweight or obesity.

Acknowledgements

We really appreciate all participants, project staff of the China Multi-Ethnic Cohort Study.

Contributions

All authors contributed significantly to this article.TC analysed the data and wrote the first version of the manuscript; WT, LC, DM and XD performed the surveys; LS and XL interpreted the results and revised the manuscript; XD and XL critically reviewed the manuscript for important intellectual content. All authors approved the final manuscript.

Funding

This work was supported by National Key R&D Program of China (grant number: 2017YFC0907303) and Chongqing medical scientific research project (grant number: 2020FYYX013).

For peer review only - http://bmjopen.bmj.com/site/about/guidelines.xhtml

Competing interests

None declared.

Patient consent

All participants had written informed consent prior to the study.

Ethics approval

The study was approved by the ethics committee of Sichuan University (No.K2016038).

Provenance and peer review

Not commissioned; externally peer reviewed.

Availability of data and materials

Our study relied on data from China Multi-Ethnic Cohort Study. The summary dataset used during the current study are available from the corresponding author on a reasonable request.

References

1. Li Y, Shen Z, Zhu B, et al. Demographic, regional and temporal trends of hyperuricemia epidemics in mainland China from 2000 to 2019: a systematic review and meta-analysis. *Glob Health Action* 2021;14(1):1874652.
2. Chen JH, Chuang SY, Chen HJ, et al. Serum uric acid level as an independent risk factor for all-cause, cardiovascular, and ischemic stroke mortality: a Chinese cohort study. *Arthritis Rheum* 2009;61(2):225-32.
3. Dehghan A, van Hoek M, Sijbrands EJ, et al. High serum uric acid as a novel risk factor for type 2 diabetes. *Diabetes Care* 2008;31(2):361-2.
4. Fang J, Alderman MH. Serum uric acid and cardiovascular mortality the NHANES I epidemiologic follow-up study, 1971-1992. National Health and Nutrition Examination Survey. *Jama* 2000;283(18):2404-10.
5. Keenan T, Blaha MJ, Nasir K, et al. Relation of uric acid to serum levels of high-sensitivity C-reactive protein, triglycerides, and high-density lipoprotein cholesterol and to hepatic

For peer review only - http://bmjopen.bmj.com/site/about/guidelines.xhtml

steatosis. *Am J Cardiol* 2012;110(12):1787-92.
6. See LC, Kuo CF, Chuang FH, et al. Hyperuricemia and metabolic syndrome: associations with chronic kidney disease. *Clin Rheumatol* 2011;30(3):323-30.
7. Sirota JC, McFann K, Targher G, et al. Elevated serum uric acid levels are associated with non-alcoholic fatty liver disease independently of metabolic syndrome features in the United States: Liver ultrasound data from the National Health and Nutrition Examination Survey. *Metabolism* 2013;62(3):392-9.
8. Gamala M, Jacobs JWG. Gout and hyperuricaemia: a worldwide health issue of joints and beyond. *Rheumatology (Oxford)* 2019;58(12):2083-85.
9. Han B, Wang N, Chen Y, et al. Prevalence of hyperuricaemia in an Eastern Chinese population: a cross-sectional study. *BMJ Open* 2020;10(5):e035614.
10. Huang XB, Zhang WQ, Tang WW, et al. Prevalence and associated factors of hyperuricemia among urban adults aged 35-79 years in southwestern China: a community-based cross-sectional study. *Sci Rep* 2020;10(1):15683.
11. Gee Teng G, Pan A, Yuan JM, et al. Cigarette Smoking and the Risk of Incident Gout in a Prospective Cohort Study. *Arthritis Care Res (Hoboken)* 2016;68(8):1135-42.
12. Han QX, Zhang D, Zhao YL, et al. Risk Factors For Hyperuricemia In Chinese Centenarians And Near-Centenarians. *Clin Interv Aging* 2019;14:2239-47.
13. Hirotsu C, Tufik S, Guindalini C, et al. Association between uric acid levels and obstructive sleep apnea syndrome in a large epidemiological sample. *PLoS One* 2013;8(6):e66891.
14. Shi T, Min M, Sun C, et al. A meta-analysis of the association between gout, serum uric acid level, and obstructive sleep apnea. *Sleep Breath* 2019;23(4):1047-57.
15. Wiener RC, Shankar A. Association between Serum Uric Acid Levels and Sleep Variables: Results from the National Health and Nutrition Survey 2005-2008. *Int J Inflam* 2012;2012:363054.
16. Xiong X, He F, Sun G, et al. The relationship between self-reported habitual snoring and hyperuricemia among Chinese urban adults: a cross-sectional study. *Sleep Med* 2020;68:207-12.
17. Zheng C, Song H, Wang S, et al. Serum Uric Acid Is Independently Associated with Risk of Obstructive Sleep Apnea-Hypopnea Syndrome in Chinese Patients with Type 2 Diabetes. *Dis Markers* 2019;2019:4578327.
18. Zhao X, Hong F, Yin J, et al. Cohort profile: the China Multi-Ethnic cohort (CMEC) study. *Int J Epidemiol* 2020
19. Cai Z, Xu X, Wu X, et al. Hyperuricemia and the metabolic syndrome in Hangzhou. *Asia Pac J Clin Nutr* 2009;18(1):81-7.
20. Fang S, Zhou J. Association of Daytime Napping and Diagnosed Diabetes in Middle-Aged Premenopausal, Middle-Aged Postmenopausal, and Older Postmenopausal Chinese Women. *Am J Health Promot* 2019;33(8):1107-14.
21. Xiao M, Tang X, Zhang F, et al. Association between self-reported snoring and hypertension among Chinese Han population aged 30-79 in Chongqing, China. *Environ Health Prev Med* 2020;25(1):78.
22. Swartz AM, Strath SJ, Bassett DR, Jr., et al. Estimation of energy expenditure using CSA accelerometers at hip and wrist sites. *Med Sci Sports Exerc* 2000;32(9 Suppl):S450-6.
23. van Durme C, Spaetgens B, Driessen J, et al. Obstructive sleep apnea and the risk of gout: a

For peer review only

For peer review only - http://bmjopen.bmj.com/site/about/guidelines.xhtml

population-based case-control study. *Arthritis Res Ther* 2020;22(1):92.

24. Chinese Joint Committee on the Revision of Guidelines for Prevention and Treatment of Adult Dyslipidemia. guidelines for prevention and treatment of dyslipidemia in Chinese adults （2016 version）. *Chin Circ J* 2016;31:937-53(in chinese).

25. American Diabetes Association. 2. Classification and Diagnosis of Diabetes. Diabetes Care 2017;40(Suppl 1):S11-s24.

26. Chobanian AV, Bakris GL, Black HR, et al. The Seventh Report of the Joint National Committee on Prevention, Detection, Evaluation, and Treatment of High Blood Pressure: the JNC 7 report. *Jama* 2003;289(19):2560-72.

27. Harrison R. Structure and function of xanthine oxidoreductase: where are we now? *Free Radic Biol Med* 2002;33(6):774-97.

28. Gozal D, Kheirandish L. Oxidant stress and inflammation in the snoring child: confluent pathways to upper airway pathogenesis and end-organ morbidity. *Sleep Med Rev* 2006;10(2):83-96.

29. Puig F, Rico F, Almendros I, et al. Vibration enhances interleukin-8 release in a cell model of snoring-induced airway inflammation. *Sleep* 2005;28(10):1312-6.

30. Real JT, Martínez-Hervás S, García-García AB, et al. Circulating mononuclear cells nuclear factor-kappa B activity, plasma xanthine oxidase, and low grade inflammatory markers in adult patients with familial hypercholesterolaemia. *Eur J Clin Invest* 2010;40(2):89-94.

31. Zanetti M, Zenti M, Barazzoni R, et al. HELP LDL apheresis reduces plasma pentraxin 3 in familial hypercholesterolemia. *PLoS One* 2014;9(7):e101290.

32. Ma J, Zhang H, Wang H, et al. Association Between Self-Reported Snoring and Metabolic Syndrome: A Systematic Review and Meta-Analysis. *Front Neurol* 2020;11:517120.

33. Zhang N, Chen Y, Chen S, et al. Self-Reported Snoring Is Associated with Dyslipidemia, High Total Cholesterol, and High Low-Density Lipoprotein Cholesterol in Obesity: A Cross-Sectional Study from a Rural Area of China. *Int J Environ Res Public Health* 2017;14(1)

34. Dai N, Shi Q, Hua Y, et al. Snoring frequency and risk of type 2 diabetes mellitus: a prospective cohort study. *BMJ Open* 2021;11(5):e042469.

35. Lee SK, Choi K, Chang YH, et al. Increased risk for new-onset hypertension in midlife male snorers: The 14-year follow-up study. *J Sleep Res* 2019;28(5):e12757.

36. Chang HJ, Lin KR, Lin MT, et al. Associations Between Lifestyle Factors and Reduced Kidney Function in US Older Adults: NHANES 1999-2016. *Int J Public Health* 2021;66:1603966.

37. Cui LF, Shi HJ, Wu SL, et al. Association of serum uric acid and risk of hypertension in adults: a prospective study of Kailuan Corporation cohort. *Clin Rheumatol* 2017;36(5):1103-10.

38. Peppard PE, Ward NR, Morrell MJ. The impact of obesity on oxygen desaturation during sleep-disordered breathing. *Am J Respir Crit Care Med* 2009;180(8):788-93.

39. Qi D, Liu J, Wang C, et al. Sex-specific differences in the prevalence of and risk factors for hyperuricemia among a low-income population in China: a cross-sectional study. *Postgrad Med* 2020;132(6):559-67.

40. Chen Y, Xia Y, Han X, et al. Association between serum uric acid and atrial fibrillation: a cross-sectional community-based study in China. *BMJ Open* 2017;7(12):e019037.

41. Qiu L, Cheng XQ, Wu J, et al. Prevalence of hyperuricemia and its related risk factors in healthy adults from Northern and Northeastern Chinese provinces. *BMC Public Health* 2013;13:664.

For peer review only - http://bmjopen.bmj.com/site/about/guidelines.xhtml

42. Jennum P, Hein HO, Suadicani P, et al. Cardiovascular risk factors in snorers. A cross-sectional study of 3,323 men aged 54 to 74 years: the Copenhagen Male Study. *Chest* 1992;102(5):1371-6.

For peer review only

For peer review only - http://bmjopen.bmj.com/site/about/guidelines.xhtml

Figure 1 Data cleaning flow chart

For peer review only

For peer review only - http://bmjopen.bmj.com/site/about/guidelines.xhtml

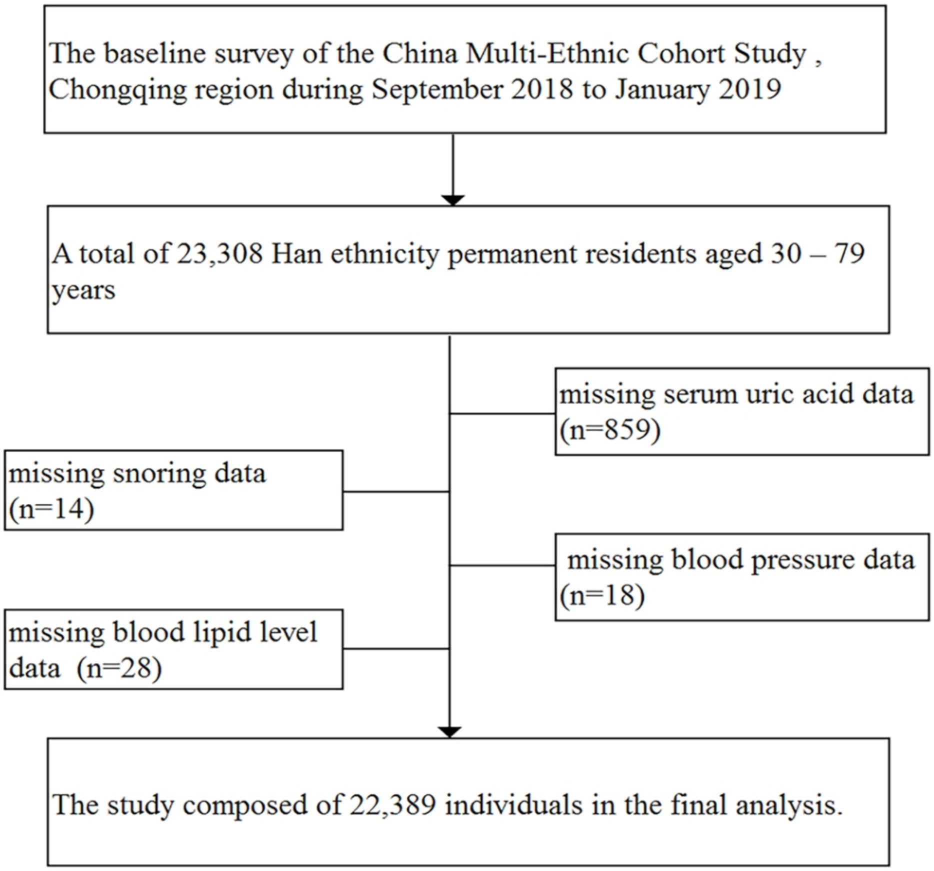

Figure 1 Data cleaning flow chart

101x101mm (300 x 300 DPI)

STROBE Statement—Checklist of items that should be included in reports of ***cross-sectional studies***

	Item No	Recommendation	Page No
**Title and abstract**	1	(*a*) Indicate the study's design with a commonly used term in the title or the abstract	1
		(*b*) Provide in the abstract an informative and balanced summary of what was done and what was found	2-3
**Introduction**			
Background/rationale	2	Explain the scientific background and rationale for the investigation being reported	3-4
Objectives	3	State specific objectives, including any prespecified hypotheses	4
**Methods**			
Study design	4	Present key elements of study design early in the paper	4
Setting	5	Describe the setting, locations, and relevant dates, including periods of recruitment, exposure, follow-up, and data collection	4-5
Participants	6	(*a*) Give the eligibility criteria, and the sources and methods of selection of participants	5
Variables	7	Clearly define all outcomes, exposures, predictors, potential confounders, and effect modifiers. Give diagnostic criteria, if applicable	5-8
Data sources/ measurement	8*	For each variable of interest, give sources of data and details of methods of assessment (measurement). Describe comparability of assessment methods if there is more than one group	5-8
Bias	9	Describe any efforts to address potential sources of bias	4
Study size	10	Explain how the study size was arrived at	5
Quantitative variables	11	Explain how quantitative variables were handled in the analyses. If applicable, describe which groupings were chosen and why	5-8
Statistical methods	12	(*a*) Describe all statistical methods, including those used to control for confounding	8
		(*b*) Describe any methods used to examine subgroups and interactions	8
		(*c*) Explain how missing data were addressed	5
		(*d*) If applicable, describe analytical methods taking account of sampling strategy	
		(*e*) Describe any sensitivity analyses	
**Results**			
Participants	13*	(a) Report numbers of individuals at each stage of study—eg numbers potentially eligible, examined for eligibility, confirmed eligible, included in the study, completing follow-up, and analysed	5,9
		(b) Give reasons for non-participation at each stage	5
		(c) Consider use of a flow diagram	5
Descriptive data	14*	(a) Give characteristics of study participants (eg demographic, clinical, social) and information on exposures and potential confounders	9-11
		(b) Indicate number of participants with missing data for each variable of interest	
Outcome data	15*	Report numbers of outcome events or summary measures	9
Main results	16	(*a*) Give unadjusted estimates and, if applicable, confounder-adjusted estimates and their precision (eg, 95% confidence interval). Make clear which confounders were adjusted for and why they were included	11-14

For peer review only - http://bmjopen.bmj.com/site/about/guidelines.xhtml

		(*b*) Report category boundaries when continuous variables were categorized	5-8
		(*c*) If relevant, consider translating estimates of relative risk into absolute risk for a meaningful time period	
Other analyses	17	Report other analyses done—eg analyses of subgroups and interactions, and sensitivity analyses	11-14
**Discussion**			
Key results	18	Summarise key results with reference to study objectives	14
Limitations	19	Discuss limitations of the study, taking into account sources of potential bias or imprecision. Discuss both direction and magnitude of any potential bias	18
Interpretation	20	Give a cautious overall interpretation of results considering objectives, limitations, multiplicity of analyses, results from similar studies, and other relevant evidence	14-17
Generalisability	21	Discuss the generalisability (external validity) of the study results	18
**Other information**			
Funding	22	Give the source of funding and the role of the funders for the present study and, if applicable, for the original study on which the present article is based	19

*Give information separately for exposed and unexposed groups.

Note: An Explanation and Elaboration article discusses each checklist item and gives methodological background and published examples of transparent reporting. The STROBE checklist is best used in conjunction with this article (freely available on the Web sites of PLoS Medicine at http://www.plosmedicine.org/, Annals of Internal Medicine at http://www.annals.org/, and Epidemiology at http://www.epidem.com/). Information on the STROBE Initiative is available at www.strobe-statement.org.

For peer review only

For peer review only - http://bmjopen.bmj.com/site/about/guidelines.xhtml

BMJ Open

Association of self-reported snoring and hyperuricemia: a large cross-sectional study in Chongqing, China

Journal:	*BMJ Open*
Manuscript ID	bmjopen-2021-056143.R2
Article Type:	Original research
Date Submitted by the Author:	28-Feb-2022
Complete List of Authors:	chen, ting; Chongqing Medical University, School of Public Health and Management, Research Center for Medicine and Social Development,; Chongqing Municipal Center for Disease Control and Prevention<br>Ding, Xianbin; Chongqing Municipal Center for Disease Control and Prevention<br>Tang, Wenge; Chongqing Municipal Center for Disease Control and Prevention<br>Chen, Liling; Chongqing Municipal Center for Disease Control and Prevention<br>Mao, Deqiang; Chongqing Municipal Center for Disease Control and Prevention<br>Song, Lingling; Chongqing Medical University, School of Public Health and Management,Research Center for Medicine and Social Development,<br>Lian, Xuemei; Chongqing Medical University, School of Public Health and Management,Research Center for Medicine and Social Development,
**Primary Subject Heading**:	Public health
Secondary Subject Heading:	Epidemiology
Keywords:	EPIDEMIOLOGY, PREVENTIVE MEDICINE, PUBLIC HEALTH, Other metabolic, e.g. iron, porphyria < DIABETES & ENDOCRINOLOGY

SCHOLARONE™
Manuscripts

I, the Submitting Author has the right to grant and does grant on behalf of all authors of the Work (as defined in the below author licence), an exclusive licence and/or a non-exclusive licence for contributions from authors who are: i) UK Crown employees; ii) where BMJ has agreed a CC-BY licence shall apply, and/or iii) in accordance with the terms applicable for US Federal Government officers or employees acting as part of their official duties; on a worldwide, perpetual, irrevocable, royalty-free basis to BMJ Publishing Group Ltd ("BMJ") its licensees and where the relevant Journal is co-owned by BMJ to the co-owners of the Journal, to publish the Work in this journal and any other BMJ products and to exploit all rights, as set out in our licence.

The Submitting Author accepts and understands that any supply made under these terms is made by BMJ to the Submitting Author unless you are acting as an employee on behalf of your employer or a postgraduate student of an affiliated institution which is paying any applicable article publishing charge ("APC") for Open Access articles. Where the Submitting Author wishes to make the Work available on an Open Access basis (and intends to pay the relevant APC), the terms of reuse of such Open Access shall be governed by a Creative Commons licence – details of these licences and which Creative Commons licence will apply to this Work are set out in our licence referred to above.

Other than as permitted in any relevant BMJ Author's Self Archiving Policies, I confirm this Work has not been accepted for publication elsewhere, is not being considered for publication elsewhere and does not duplicate material already published. I confirm all authors consent to publication of this Work and authorise the granting of this licence.

For peer review only

Title: Association of self-reported snoring and hyperuricemia: a large cross-sectional study in Chongqing, China

Authors: Ting Chen[1,2], Xianbin Ding[2] ,Wenge Tang[2], Liling Chen[2], Deqiang Mao[2], Lingling Song[1] ,Xuemei Lian[1*]

Affiliations:

1 Research Center for Medicine and Social Development, School of Public Health and Management, Chongqing Medical University, Chongqing, China

2 Chongqing Center for Disease Control and Prevention, Chongqing, China

*** Corresponding authors:**

Xuemei Lian, No.1 Yixueyuan Rd, Yuzhong District, Chongqing 400016, China, Email: xuemeilian@cqmu.edu.cn

For peer review only

For peer review only - http://bmjopen.bmj.com/site/about/guidelines.xhtml

Abstract

Objective: To examine the relationship between self-reported snoring and hyperuricemia in a large-scale population in Chongqing, China.

Setting: Face-to-face electronic questionnaire survey, physical examination and biological sample testing were conducted in 13 districts of Chongqing. Chongqing is a municipality in southwest China.

Participants: In this study, 23,308 Han ethnicity permanent residents aged 30 – 79 years were recruited. Individuals missing data were excluded, 22,389 subjects were included in final analysis.

Primary and secondary outcome measures: Serum uric acid (UA) was measured using an oxidase method. Hyperuricemia was defined as serum UA >420 µmol/L in men and >360 µmol/L in women. Information about self-reported snoring was obtained by questionnaire survey. All participants were divided into "no snoring" "snoring occasionally" and "snoring frequently". Multivariable logistic regression analysis was performed to assess the relationship between self-reported snoring and hyperuricemia.

Results: The prevalence of hyperuricemia was 14.43%, and snorers were more likely to have hyperuricemia than non-snorer in different age and gender groups. For the total population, those who snore occasionally or frequently were more likely to be hyperuricemia (OR 1.19, 95% CI 1.07-1.31; OR 1.33, 95% CI 1.19-1.47) compared with no snoring people. Stratification by age, gender and body mass index (BMI), we found that the positive association between snoring frequently and hyperuricemia was

For peer review only - http://bmjopen.bmj.com/site/about/guidelines.xhtml

insisted in different age, gender and high BMI groups, and the strength of association varied with different age, gender and BMI category.

Conclusion: Snoring frequency was positively associated with higher risk of hyperuricemia. Snoring frequently may be a signal for hyperuricemia, especially for females, those over 59 years of age, or those who are overweight or obese.

Keywords: Self-reported snoring, Hyperuricemia, Chinese Han population

Strengths and limitations of this study:

The sample size of the study was relatively large.

This study analysis was thorough and comprehensive.

The cross-sectional data analysis limited the ability to explore the casual relationship.

Our study results were limited by age and ethnicity.

Snoring frequency data was subjective.

Introduction

Hyperuricemia is a common chronic disease caused by purines metabolism dysfunction and uric acid excretion disorder. Hyperuricemia is becoming an important public health issue with its increasing prevalence in China. A meta-analysis study including 177 eligible studies between 2000 and 2019 reported that the prevalence of hyperuricemia was 16.4% in mainland China [1]. Previous studies have confirmed that hyperuricemia not only is associated with the development of gout, but also increases risks of vascular diseases, diabetes mellitus, kidney disease, and metabolic syndrome [2-7]. Given the large disease burden related to hyperuricemia [8], it is necessary to recognize more risk factors correlated with it.

Meanwhile, various major risk factors for hyperuricemia have been found, such as

For peer review only

low economic status [9], obesity [10],and smoking [11], and some studies have found a link between sleep-disordered breathing and hyperuricemia [12-17]. However, the association of snoring and hyperuricemia has been studied much less extensively. To our knowledge, only three studies [12 15 16] have been referred to explore the relationship of snoring and hyperuricemia. In addition, the three studies had small sample sizes, considered confounding factors insufficiently and did not examine the association by age or body mass index (BMI) category stratified analysis.

This study aimed to examine comprehensively the relationship of self-reported snoring and hyperuricemia after controlling for potential confounding factors in a large-scale population in Chongqing, China.

Methods

Participants

The present study was from the baseline survey of the China Multi-Ethnic Cohort Study [18], Chongqing region, which is a collaborated study by Sichuan University, Chongqing Medical University and Chongqing Center for Disease Control and Prevention. This study was carried out in 13 districts of Chongqing, including Yuzhong District, Jiulongpo District, Nan·an District, Ba·nan District, Changshou District, Jiangjin District, Hechuan District, Qijiang District, Dazu District, Tongnan District, Rongchang District, Wulong District, Fengdu District. In brief, a face-to-face electronic questionnaire survey, physical examination and biological sample testing were conducted in this study. In order to ensure the quality of research, strict quality control measures were applied, for example checking electronic recording. A total of

For peer review only - http://bmjopen.bmj.com/site/about/guidelines.xhtml

23,308 Han ethnicity permanent residents aged 30 – 79 years were recruited by using randomized sampling during September 2018 to January 2019. The sample inclusion criteria and exclusion criteria have been described in detail previously [18]. We excluded individual data missing serum uric acid (n=859) or snoring (n=14) or blood pressure (n=18) or blood lipid level (n=28). Finally, this present study consisted of 22,389 (figure 1) individuals providing complete information. All participants had written informed consent prior to the study. This study was approved by the ethics committee of Sichuan University (No.K2016038).

Measures

Serum uric acid (UA) measurement and the definition of hyperuricemia

After fasting for 12 hours, venous blood samples were collected from all participants. Within 2 hours after collection, blood samples were transported in dry ice to one medical laboratory certified by Chongqing Center for Disease Control and Prevention. Serum UA was measured with a Beckman Coulter AU 680 device using an oxidase method in Chongqing Di·an Medical Laboratory Center. The validity of serum UA assessment was described by relative deviation, which was controlled within 10%. The coefficient of variations of serum UA assessment was ≤4.0% within the group. Hyperuricemia was defined as serum UA >420 μmol/L in men and >360 μmol/L in women [9 19].

Measurement of snoring

Information about self-reported snoring was obtained by the question “Do you snore when you have a sleep?” Then, the frequency of snoring (“occasionally” and

For peer review only - http://bmjopen.bmj.com/site/about/guidelines.xhtml

“frequently”) was asked for those who answered “yes”. Finally, all participants were divided into “no snoring” “snoring occasionally” and “snoring frequently”.

Assessment of covariates

The present study included sociodemographic characteristics such as age, gender (male, female), marital status, education level, residential region (urban, rural), employment status (yes, no), household yearly income level (＜60,000 RMB,⩾ 60,000 RMB). Consistent with previous studies in China [20 21], participates were grouped into low-aged adults (＜45 years), middle-aged adults (45-59 years) and older adults (＞59 years). Marital status was categorized into married or living with partner and others, including widowed, unmarried, divorced or separated. Education level was classified into primary or below (elementary school or illiterate), secondary (junior school, senior school or secondary vocational school) and college or above.

Moreover, the present study collected information about health-related behaviours. Smoking status was grouped into never smoker, current smoker and ever smoker. Alcohol drinking status was categorized into never or hardly drinker, occasionally drinker (only drink on special occasions or the frequency less than once a week), often drinker (at least once a week in the past year). Tea and sugar-sweetened beverage drinking status was defined by the question “Have you ever been drinking tea/ sugar-sweetened beverage weekly for more than half a year?” Accordingly, participants were divided into drinker and non-drinker. Oil and salt intake were collected by asking about the consumption of oil and salt per month in the past year. Energy intake was measured using a semi-quantitative standardized food frequency

For peer review only - http://bmjopen.bmj.com/site/about/guidelines.xhtml

questionnaire with 13 food categories, including red meat, aquatic/sea food products and so on. Red meat intake was transformed into four categories (≤210.00, 210.01-350.00, 350.01-700.00, ＞700.00 g/week) according to its quartiles. Similarly aquatic/sea food products intake was also transformed into four categories (≤15.17, 15.18-93.33, 93.34-200.00, ＞200.00 g/week) according to its quartiles. Physical activity level (expressed as metabolic equivalent, MET) was estimated based on self-reported activities using a Compendium of Physical Activities [22], and the physical activities questionnaire referenced the global physical activity questionnaire. Nocturnal sleep and midday nap duration were collected according to the two questions: "How many hours do you usually nocturnal sleep every day in the past month ?" and "How many minutes do you usually midday nap every day in the past month ?"

Lastly, the information regarding clinic variables were obtained including BMI, kidney function, dyslipidaemia, diabetes and hypertension. BMI was calculated as weight divided by height^2 (kg/m^2) and grouped into "<24" "24 - 27.9" and "≥28" [16]. Kidney function was stratified 5 stages by estimated glomerular filtration (eGFR): Chronic kidney disease 1(CKD 1) (eGFR≥90 ml/min), CKD 2 (eGFR 60–89 ml/min), CKD 3(eGFR 30–59 ml/min), CKD 4 (eGFR 15–29 ml/min), CKD 5 (eGFR ＜15 ml/min) and eGFR was estimated by serum creatinine with the abbreviated Modification of Diet in Renal Disease equation (MDRD) formula ($175\times$ (serum creatinine(μmol/L)/88.4)$^{-1.234}$ $\times$ (age)$^{-0.179}$ $\times$ (0.79 if female))[23] . Dyslipidaemia was defined using serum total cholesterol concentration of ≥6.2mmol/l, triacylglycerol

For peer review only - http://bmjopen.bmj.com/site/about/guidelines.xhtml

concentration of ⩾2.3 mmol/l, low-density lipoprotein cholesterol concentration of ⩾4.1 mmol/l, high-density lipoprotein cholesterol concentration of < 1.0 mmol/l or a self-reported diagnosis of dyslipidaemia [24]. Diabetes was diagnosed as fasting blood glucose level of ⩾7.0 mmol/l, glycosylated haemoglobin percentage of ⩾6.5% or a self-reported diagnosis of diabetes[25]. Participants with were regarded as having hypertension if any of the following three items were met: systolic blood pressure of ⩾140 mmHg, diastolic blood pressure of ⩾90 mmHg or self-reported diagnosis of hypertension[26].

Statistical analysis

The analyses were performed with the SPSS, version 25.0. Characteristics of the participants, including sociodemographic characteristics, health-related behaviours and clinic variables, were compared according to the frequency of snoring ("no", "occasionally" and "frequently"). All continuous data were described as mean ± standard deviations, while categorical data were summarized as percentages. The one-way analysis of variance was used to compare the difference in continuous data, and chi-square test was used to compare the difference in categorical data. Then, the prevalence of hyperuricemia with different snoring frequency was examined using the chi-square test in different age and gender groups. Finally, Multivariable logistic regression analysis was performed to evaluate the relationship between snoring and hyperuricemia after adjusting for different covariates in different age groups, gender groups , BMI groups and total population, and odds ratio (OR) and 95% confidence interval (CI) for the outcome variable were calculated. Two-sided *P* value of <0.05

For peer review only - http://bmjopen.bmj.com/site/about/guidelines.xhtml

was considered to be statistically significant.

Patient and public involvement

Patients and the public were not involved in the design of the study, recruitment and conduct of the study or dissemination of the study results.

Results

Sample characteristics of participants

The characteristics of participants based on the categories of self-reported snoring are showed in Table 1.The study composed of 22,389 individuals in the final analysis. Of the study subjects, 51.23%, 26.71%, 22.06% reported “no snoring”, “snoring occasionally”, “snoring frequently”, respectively. Participants who snore were more likely to be aged 45–59, male, employed person, non-smokers, occasionally alcohol drinkers, non-tea drinkers. In addition, compared with those who reported “no snoring”, snorers had a higher level of energy take, midday nap duration, BMI, dyslipidaemia, diabetes, hypertension, but had a lower level of physical activity, nocturnal sleep duration.

Table 1 Characteristics of participants based on the categories of self-reported snoring

Characteristics	Self-reported snoring			*P* value
	No	Occasionally	Frequently	
Number of subjects	11469	5981	4939	
**Sociodemographic Characteristics**				
Age				< 0.001
< 45	36.18	30.16	19.80	

For peer review only - http://bmjopen.bmj.com/site/about/guidelines.xhtml

45-59	39.64	42.37	43.59	
＞59	24.18	27.47	36.61	
Male	35.26	53.02	66.13	＜0.001
Married or living with partner	87.75	88.13	88.01	0.742
Education level				＜0.001
Primary or below	33.16	28.57	36.83	
Secondary	49.94	53.10	49.20	
College or above	16.90	18.32	13.97	
Urban	27.49	33.42	25.81	＜0.001
Employment	63.05	62.43	59.73	
Income (＜60,000 RMB)	59.51	55.12	59.91	＜0.001
**Health-related behaviours**				
Smoking				＜0.001
Never	80.99	69.27	59.71	
Current	14.61	23.61	31.00	
Ever	4.40	7.12	9.29	
Alcohol drinking				＜0.001
Never or hardly	53.00	38.40	37.84	
Occasionally	37.56	45.23	40.27	
Often	9.44	16.37	21.89	
Tea drinker	16.27	24.33	28.10	＜0.001
Sugar-sweetened beverage drinker	1.96	2.51	3.52	＜0.001
Oil intake (g/week)	56.71±0.30	56.66±0.42	58.33+0.47	0.008
Salt intake (g/week)	48.36±0.27	47.45±0.36	51.04±0.42	＜0.001
Energy intake (kcal/week)	12776.57±39.74	13241.66±56.36	13838.48±64.34	＜0.001
Red meat intake (g/week)				＜0.001
≤210.00	27.32	25.73	23.14	
210.01-350.00	26.06	25.10	21.83	
350.01-700.00	30.23	30.48	31.91	

For peer review only

For peer review only - http://bmjopen.bmj.com/site/about/guidelines.xhtml

＞700.00	16.39	18.69	23.12	
Aquatic/sea food products intake (g/week）				< 0.001
≤15.17	27.10	22.07	23.87	
15.18-93.33	25.01	25.75	23.73	
93.34-200.00	25.38	26.25	24.13	
＞200.00	22.51	25.93	28.26	
Physical activity (MET minutes /week)	10176.77±63.98	9731.86±86.57	9777.99±101.00	< 0.001
Nocturnal sleep duration (h/day)	7.18±0.01	7.04±0.02	6.97±0.02	< 0.001
Midday nap duration (min/day)	28.91±0.33	31.17±0.45	33.43±0.54	< 0.001
**Clinic variables**				
BMI ((kg/m^2))				< 0.001
＜24	55.07	39.26	24.20	
24-27.9	35.91	45.81	47.99	
≥28	9.02	14.93	27.82	
Kidney function				
CKD1	84.33	80.45	75.40	< 0.001
CKD2	14.97	18.66	23.16	
CKD3	0.66	0.80	1.28	
CKD4	0.02	0.05	0.12	
CKD5	0.02	0.03	0.04	
Dyslipidaemia	23.99	33.46	44.26	< 0.001
Diabetes	7.35	10.18	15.02	< 0.001
Hypertension	28.13	36.23	50.50	< 0.001

Continuous data were described as mean± standard deviations, and statistical significance was assessed by the one-way analysis of variance.

Categorical data were summarized as percentages (%), and statistical significance was assessed by chi-square test.

The prevalence of hyperuricemia

The prevalence of hyperuricemia according to different snoring frequency with different groups is presented in Table 2. In all, the prevalence of hyperuricemia was

For peer review only - http://bmjopen.bmj.com/site/about/guidelines.xhtml

For peer review only

14.43% (3230/22389) in our study population. Compared with the "no snoring" group, the prevalence of hyperuricemia was higher in the "snoring occasionally" and "snoring frequently" groups among the three age groups (P<0.05). In addition, those who reported "snoring occasionally" or "snoring frequently" were more likely to have hyperuricemia than "no snoring" group in different gender groups (P<0.05). Similarly, in the total population, higher prevalence of hyperuricemia was found in the "snoring occasionally" or "snoring frequently" compared with "no snoring" group.

Table 2 Prevalence of hyperuricemia according to snoring frequency in different groups

Snoring frequency	Age [*n* (%)]			Gender [*n* (%)]		Total
	< 45	45-59	> 59	Male	Female	
No	376(9.06)	441(9.70)	358(12.91)	650(16.07)	525(7.07)	1175(10.25)
Occasionally	326(18.07)	370(14.60)	261(15.89)	659(20.78)	298(10.60)	957(16.00)
Frequently	299(30.57)	452(20.99)	347(19.19)	820(25.11)	278(16.62)	1098(22.23)
χ2	322.34	160.32	33.23	91.72	156.38	418.14
P value	< 0.001	< 0.001	< 0.001	< 0.001	< 0.001	< 0.001

Association between self-reported snoring and hyperuricemia

Results of multivariable logistic regression analysis about the association between self-reported snoring and hyperuricemia are showed in Table 3. In the total population, after adjusting for sociodemographic characteristics, health-related behaviours and clinic variables, those who reported "snoring occasionally" or "snoring frequently" were more likely to be hyperuricemia (OR 1.19, 95%CI 1.07-1.31; OR 1.33, 95%CI 1.19-1.47, respectively) compared with "no snoring" group. The stratified analysis by age results showed: after adjusting for all variables, in three age groups higher odd

For peer review only - http://bmjopen.bmj.com/site/about/guidelines.xhtml

ratio of hyperuricemia (OR 1.32, 95%CI 1.07-1.63; OR 1.23, 95%CI 1.04-1.45; OR 1.33, 95%CI 1.11-1.58, respectively) was found in the "snoring frequently" group compared with "no snoring" group. The stratified analysis by gender results showed: after adjusting for all variables, those who reported "snoring frequently" had a higher odd ratio of hyperuricemia in male (OR 1.24, 95%CI 1.08-1.41) and female (OR 1.33, 95%CI 1.12-1.59, respectively) than "no snoring" group. The stratified analysis by BMI results showed: after adjusting for all variables, those who reported "snoring frequently" remained at an increased risk of hyperuricemia in BMI 24-27.9 and ⩾28 kg/m^2 groups (OR 1.35, 95%CI 1.16-1.56; OR 1.41, 95%CI 1.15-1.73), but the association was not found in BMI ＜24 kg/m^2 group (*P*>0.05).

Table 3 Association between self-reported snoring and hyperuricemia

Variables	NO	Occasionally		Frequently	
	OR（95%CI）	OR（95%CI）	*P* value	OR（95%CI）	*P* value
**Age**					
**< 45**					
Model 1	1.00(reference)	2.21(1.89,2.60)	< 0.001	4.42(3.72,5.25)	< 0.001
Model 2	1.00(reference)	1.51(1.28,1.79)	< 0.001	2.44(2.03,2.94)	< 0.001
Model 3	1.00(reference)	1.49(1.26,1.77)	< 0.001	2.38(1.97,2.88)	< 0.001
Model 4	1.00(reference)	1.16(0.96,1.39)	0.13	**1.32(1.07,1.63)**	**0.01**
**45-59**					
Model 1	1.00(reference)	1.59(1.37,1.85)	< 0.001	2.47(2.14,2.85)	< 0.001
Model 2	1.00(reference)	1.33(1.14,1.55)	< 0.001	1.88(1.62,2.18)	< 0.001
Model 3	1.00(reference)	1.29(1.11,1.50)	0.00	1.83(1.57,2.12)	< 0.001

For peer review only - http://bmjopen.bmj.com/site/about/guidelines.xhtml

Model 4	1.00(reference)	1.09(0.92,1.28)	0.32	**1.23(1.04,1.45)**	**0.02**
**> 59**					
Model 1	1.00(reference)	1.28(1.07,1.52)	0.01	1.60(1.36,1.88)	< 0.001
Model 2	1.00(reference)	1.25(1.05,1.48)	0.01	1.59(1.35,1.87)	< 0.001
Model 3	1.00(reference)	1.22(1.03,1.46)	0.03	1.58(1.34,1.87)	< 0.001
Model 4	1.00(reference)	1.18(0.98,1.42)	0.09	**1.33(1.11,1.58)**	**0.00**
**Gender**					
**Male**					
Model 1	1.00(reference)	1.37(1.22,1.54)	< 0.001	1.75(1.56,1.96)	< 0.001
Model 2	1.00(reference)	1.30(1.15,1.47)	< 0.001	1.79(1.59,2.00)	< 0.001
Model 3	1.00(reference)	1.25(1.11,1.41)	< 0.001	1.71(1.52,1.93)	< 0.001
Model 4	1.00(reference)	1.08(0.94,1.23)	0.28	**1.24(1.08,1.41)**	**0.00**
**Female**					
Model 1	1.00(reference)	1.56(1.34,1.81)	< 0.001	2.62(2.24,3.06)	< 0.001
Model 2	1.00(reference)	1.42(1.22,1.65)	< 0.001	2.13(1.81,2.50)	< 0.001
Model 3	1.00(reference)	1.43(1.22,1.66)	< 0.001	2.13(1.81,2.51)	< 0.001
Model 4	1.00(reference)	**1.21(1.03,1.42)**	**0.02**	**1.33(1.12,1.59)**	**0.00**
**BMI**					
**＜24**					
Model 1	1.00(reference)	1.48(1.25,1.76)	< 0.001	1.96(1.61,2.40)	< 0.001
Model 2	1.00(reference)	1.22(1.02,1.45)	0.03	1.39(1.12,1.71)	0.00
Model 3	1.00(reference)	1.20(1.00,1.43)	0.05	1.36(1.10,1.68)	0.00
Model 4	1.00(reference)	1.13(0.94,1.36)	0.19	1.16(0.93,1.45)	0.19
**24-27.9**					
Model 1	1.00(reference)	1.43(1.25,1.64)	< 0.001	1.84(1.61,2.10)	< 0.001
Model 2	1.00(reference)	1.21(1.05,1.39)	0.01	1.50(1.30,1.73)	< 0.001
Model 3	1.00(reference)	1.19(1.03,1.37)	0.02	1.48(1.28,1.70)	< 0.001

For peer review only

For peer review only - http://bmjopen.bmj.com/site/about/guidelines.xhtml

Model 4	1.00(reference)	1.15(0.99,1.33)	0.06	**1.35(1.16,1.56)**	**< 0.001**
**⩾28**					
Model 1	1.00(reference)	1.42(1.16,1.74)	0.00	1.68(1.40,2.02)	< 0.001
Model 2	1.00(reference)	1.30(1.06,1.61)	0.01	1.47(1.22,1.78)	< 0.001
Model 3	1.00(reference)	1.30 (1.05,1.61)	0.02	1.47(1.21,1.79)	< 0.001
Model 4	1.00(reference)	1.25(1.00,1.55)	0.05	**1.41(1.15,1.73)**	**0.00**
**Total**					
Model 1	1.00(reference)	1.67(1.52,1.83)	< 0.001	2.50(2.29,2.74)	< 0.001
Model 2	1.00(reference)	1.45(1.32,1.59)	< 0.001	2.03(1.84,2.23)	< 0.001
Model 3	1.00(reference)	1.43(1.30,1.57)	< 0.001	2.00(1.81,2.20)	< 0.001
Model 4	1.00(reference)	**1.19(1.07,1.31)**	**0.00**	**1.33(1.19,1.47)**	**<0.001**

Model 1: unadjusted model
Model 2: adjusted for gender (when stratification by gender, it was excluded), age (when stratification by age, it was excluded), marital status, education level, residential region, employment status and household yearly income level
Model 3: additionally adjusted for smoking status, alcohol drinking status, tea drinking status, sugar-sweetened beverage drinking status, oil intake, salt intake, energy intake, red meat intake, aquatic/sea food products intake, physical activity level, nocturnal sleep and midday nap duration
Model 4: additionally BMI, kidney function, dyslipidaemia, diabetes and hypertension

Discussion

In our study, we found that self-reported snoring was independently associated with hyperuricemia in total population. After adjusting for the sociodemographic characteristics, health-related behaviours and clinic variables, those who reported “snoring occasionally” and “snoring frequently” had 19% and 33% increased risk of hyperuricemia compared with “no snoring” group, respectively.

Our findings are consistent with those results of previous studies. An analysis of 6491 participants from the National Health and Nutrition Survey 2005–2008 found an

For peer review only - http://bmjopen.bmj.com/site/about/guidelines.xhtml

For peer review only

increased risk of hyperuricemia associated with snoring, which found similar results with our study[15]. Xiong et al. enrolled 7699 Chinese urban adults from Nanjing between September 2016 and February 2018, they verified the positive link between snoring and hyperuricemia [16]. A study of data from the baseline survey of the China Hainan Centenarian Cohort Study (CHCCS) also showed a positive association between snoring and hyperuricemia in centenarians [12]. The possible mechanisms can partly explain the positive association between snoring and hyperuricemia. Snoring is often accompanied by oxygen desaturation leading to tissue hypoxia, and then this accelerates the breakdown of adenosine triphosphate, resulting in the accumulation of uric acid from purine catabolic product by xanthine dehydrogenase and xanthine oxidase [27]. In addition, previous study has showed that vibration induced by snoring is related to inflammation response [28], otherwise some studies have found the association of inflammation response and metabolic disease, for example dyslipidaemia [29 30]. Therefore, whether the effect of snoring on hyperuricemia is mediated by inflammatory pathway needs to be further exploration.

Compared with previous studies, our study conducted more detailed relationship analysis of snoring and hyperuricemia in different age and gender groups. To our knowledge, no studies have been analysed age differences of association between snoring and hyperuricemia. We found that those who snore frequently with aged >59 year old had a 33% increased risk of hyperuricemia ($P < 0.05$) compared with no snoring people, and the strength of association was stronger than age 45- 59 (23%) and aged <45 (32%) groups, which might be related to decreasing metabolic function

For peer review only - http://bmjopen.bmj.com/site/about/guidelines.xhtml

significantly in old people. This result suggested that people over 59 who snore frequently should pay more attention to their uric acid level in order to achieve early prevention of gout. In the present study, for male and female, those who snore frequently had a 24% and 33% increased risk of hyperuricemia ($P<0.05$) than no snoring people, respectively. Our findings showed the strength of association in female was stronger compared with male, which are consistent with previous study [16]. To the best of our knowledge, no study has explored the possible mechanisms. It might be related to hormones difference in female and male. Therefore, more evidence studies on the mechanism of different strength relationship between snoring and hyperuricemia in female and male are needed. In this study, our results showed snoring occasionally and snoring frequently had increased risk of hyperuricemia than no snoring people in all different age and gender groups before adjusting for clinic variables, although the association between snoring occasionally and no snoring was not found for all groups except for female after adjusting for all confounders. This implies that BMI, kidney function, dyslipidaemia, diabetes and hypertension may affect the relationship between snoring frequency and hyperuricemia in different age and gender groups. Previous studies had confirmed snoring to be significantly associated with obesity [31], dyslipidaemia [32], diabetes [33] and hypertension [34]. In addition, those diseases may be related to serum uric acid level [35 36]. Therefore, it is necessary to adjust clinical indicators.

Besides, we conducted the stratified analysis by BMI. The result revealed that those who snore frequently with a high BMI remained at an increased risk of

For peer review only - http://bmjopen.bmj.com/site/about/guidelines.xhtml

hyperuricemia compared with no snoring people, however, the positive association did not found in the low BMI group. The difference in results of three groups indicated the necessity to stratify by BMI. And this interesting result implies that overweight or obesity plays an important role in the relationship of snoring and hyperuricemia, which may be related to hypoxemia caused by overweight or obesity [37].

In addition, our results showed that the prevalence of hyperuricemia was 14.43% in Chongqing, which was similar to the results of the Tianjin Brain Study (14.4%) [38]. However, this prevalence was more than that in the Tangshan population (13.4%) [39]. The difference may be explained by the different lifestyles and economic level in the two different areas, which are also closely related to hyperuricemia [40]. Our determined prevalence was also slightly higher than that in southwest (Chengdu and Chongqing) China hyperuricemia survey, which showed that the prevalence of hyperuricemia was 13.5% in 2013-2014[10]. This indicates that we should pay more attention to hyperuricemia due to the prevalence is increasing with the improvement of economic level and lifestyles changes.

Compared with previous study, our study is more thorough and comprehensive because the stratified analysis was conducted to explore the difference of relationship between snoring and hyperuricemia in different age, gender and BMI category groups. Only one previous study conducted a stratified analysis by gender, but this study did not consider to stratified analysis by age and BMI category [16]. Moreover, participants of the prior study were recruited from hospital, so the sample populations were not

For peer review only - http://bmjopen.bmj.com/site/about/guidelines.xhtml

representative. Relatively, community-based large samples of our study increased the representativeness and generalizability of our result to some extent. To the best of our knowledge, this is the first study to explore the association between snoring and hyperuricemia used a large sample size in southwest China. Furthermore, the present study included many potential confounders, such as smoking, alcohol drinking, tea drinking, sugar-sweetened beverage drinking, oil intake, salt intake, energy intake, red meat intake, aquatic/sea food products intake, physical activity, sleep duration, nap duration, BMI, kidney function, dyslipidaemia, diabetes and hypertension, which could minimize the bias.

The present study also has some limitations. First, our study data was a cross-sectional result from the cohort study, and the cross-sectional data analysis limited the ability to explore the casual relationship between snoring and hyperuricemia. Further follow-up data analysis could confirm whether there was causal relationship between snoring and hyperuricemia by using special statistical model. Second, snoring frequency was obtained from a subjective self-reported question, which may be different with actual snoring frequency. However, Jennum et al. had found self-reported snoring frequency was highly correlated with microphone records[41], suggesting that the self-reported snoring could be applied in scientific research. In addition, self-reported snoring manner has also been used to other similar studies of the relationship between snoring and uric acid [12 15]. Third, the present study was lack of degree of snoring, which may distort the relationship between snoring and hyperuricemia. Thus, the degree of snoring should be considered in future studies.

For peer review only - http://bmjopen.bmj.com/site/about/guidelines.xhtml

Fourth， our study was limited by age and ethnicity, so extrapolating the results to other age groups and ethnicity population should be noted.

Conclusions

In summary, snoring frequency was positively associated with higher risk of hyperuricemia in total population. In the three age groups, those who snore frequently had increased risk of hyperuricemia compared with no snoring people, and the strength of association was the strongest in age >59 group. In addition, snoring frequently is independently risk factor of hyperuricemia for male and female, and the strength of association in female was stronger compared with male. In high BMI population, those who snore frequently had increased risk of hyperuricemia compared with no snoring people, but the association was not found in BMI <24 group. Our result indicated that snoring frequently may be a signal for hyperuricemia, especially for females, those over 59 years of age, or those who are overweight or obese.

Acknowledgements

We really appreciate all participants, project staff of the China Multi-Ethnic Cohort Study.

Contributions

All authors contributed significantly to this article.TC analysed the data and wrote the first version of the manuscript; WT, LC, DM and XD performed the surveys; LS and XL interpreted the results and revised the manuscript; XD and XL critically reviewed the manuscript for important intellectual content. All authors approved the final manuscript.

For peer review only - http://bmjopen.bmj.com/site/about/guidelines.xhtml

Funding

This work was supported by National Key R&D Program of China (grant number: 2017YFC0907303) and Chongqing medical scientific research project (grant number: 2020FYYX013).

Competing interests

None declared.

Patient consent

All participants had written informed consent prior to the study.

Ethics approval

The study was approved by the ethics committee of Sichuan University (No.K2016038).

Provenance and peer review

Not commissioned; externally peer reviewed.

Availability of data and materials

Our study relied on data from China Multi-Ethnic Cohort Study. The summary dataset used during the current study are available from the corresponding author on a reasonable request.

References

1. Li Y, Shen Z, Zhu B, et al. Demographic, regional and temporal trends of hyperuricemia epidemics in mainland China from 2000 to 2019: a systematic review and meta-analysis. *Glob Health Action* 2021;14(1):1874652.
2. Chen JH, Chuang SY, Chen HJ, et al. Serum uric acid level as an independent risk factor for all-cause, cardiovascular, and ischemic stroke mortality: a Chinese cohort study. *Arthritis*

For peer review only - http://bmjopen.bmj.com/site/about/guidelines.xhtml

Rheum 2009;61(2):225-32.

3. Dehghan A, van Hoek M, Sijbrands EJ, et al. High serum uric acid as a novel risk factor for type 2 diabetes. *Diabetes Care* 2008;31(2):361-2.
4. Fang J, Alderman MH. Serum uric acid and cardiovascular mortality the NHANES I epidemiologic follow-up study, 1971-1992. National Health and Nutrition Examination Survey. *Jama* 2000;283(18):2404-10.
5. Keenan T, Blaha MJ, Nasir K, et al. Relation of uric acid to serum levels of high-sensitivity C-reactive protein, triglycerides, and high-density lipoprotein cholesterol and to hepatic steatosis. *Am J Cardiol* 2012;110(12):1787-92.
6. See LC, Kuo CF, Chuang FH, et al. Hyperuricemia and metabolic syndrome: associations with chronic kidney disease. *Clin Rheumatol* 2011;30(3):323-30.
7. Sirota JC, McFann K, Targher G, et al. Elevated serum uric acid levels are associated with non-alcoholic fatty liver disease independently of metabolic syndrome features in the United States: Liver ultrasound data from the National Health and Nutrition Examination Survey. *Metabolism* 2013;62(3):392-9.
8. Gamala M, Jacobs JWG. Gout and hyperuricaemia: a worldwide health issue of joints and beyond. *Rheumatology (Oxford)* 2019;58(12):2083-85.
9. Han B, Wang N, Chen Y, et al. Prevalence of hyperuricaemia in an Eastern Chinese population: a cross-sectional study. *BMJ Open* 2020;10(5):e035614.
10. Huang XB, Zhang WQ, Tang WW, et al. Prevalence and associated factors of hyperuricemia among urban adults aged 35-79 years in southwestern China: a community-based cross-sectional study. *Sci Rep* 2020;10(1):15683.
11. Gee Teng G, Pan A, Yuan JM, et al. Cigarette Smoking and the Risk of Incident Gout in a Prospective Cohort Study. *Arthritis Care Res (Hoboken)* 2016;68(8):1135-42.
12. Han QX, Zhang D, Zhao YL, et al. Risk Factors For Hyperuricemia In Chinese Centenarians And Near-Centenarians. *Clin Interv Aging* 2019;14:2239-47.
13. Hirotsu C, Tufik S, Guindalini C, et al. Association between uric acid levels and obstructive sleep apnea syndrome in a large epidemiological sample. *PLoS One* 2013;8(6):e66891.
14. Shi T, Min M, Sun C, et al. A meta-analysis of the association between gout, serum uric acid level, and obstructive sleep apnea. *Sleep Breath* 2019;23(4):1047-57.
15. Wiener RC, Shankar A. Association between Serum Uric Acid Levels and Sleep Variables: Results from the National Health and Nutrition Survey 2005-2008. *Int J Inflam* 2012;2012:363054.
16. Xiong X, He F, Sun G, et al. The relationship between self-reported habitual snoring and hyperuricemia among Chinese urban adults: a cross-sectional study. *Sleep Med* 2020;68:207-12.
17. Zheng C, Song H, Wang S, et al. Serum Uric Acid Is Independently Associated with Risk of Obstructive Sleep Apnea-Hypopnea Syndrome in Chinese Patients with Type 2 Diabetes. *Dis Markers* 2019;2019:4578327.
18. Zhao X, Hong F, Yin J, et al. Cohort profile: the China Multi-Ethnic cohort (CMEC) study. *Int J Epidemiol* 2020
19. Cai Z, Xu X, Wu X, et al. Hyperuricemia and the metabolic syndrome in Hangzhou. *Asia Pac J Clin Nutr* 2009;18(1):81-7.
20. Fang S, Zhou J. Association of Daytime Napping and Diagnosed Diabetes in Middle-Aged

For peer review only

For peer review only - http://bmjopen.bmj.com/site/about/guidelines.xhtml

Premenopausal, Middle-Aged Postmenopausal, and Older Postmenopausal Chinese Women. *Am J Health Promot* 2019;33(8):1107-14.

21. Xiao M, Tang X, Zhang F, et al. Association between self-reported snoring and hypertension among Chinese Han population aged 30-79 in Chongqing, China. *Environ Health Prev Med* 2020;25(1):78.
22. Swartz AM, Strath SJ, Bassett DR, Jr., et al. Estimation of energy expenditure using CSA accelerometers at hip and wrist sites. *Med Sci Sports Exerc* 2000;32(9 Suppl):S450-6.
23. Chinese eGFR Investigation Collaboration. Modification and evaluation of MDRD estimating equation for Chinese patients with chronic kidney disease. Chin J Nephrol 2006;22(10):589-95(in Chinese).
24. Chinese Joint Committee on the Revision of Guidelines for Prevention and Treatment of Adult Dyslipidemia. Guidelines for prevention and treatment of dyslipidemia in Chinese adults（2016 version）. *Chin Circ J* 2016;31:937-53(in Chinese).
25. American Diabetes Association. 2. Classification and Diagnosis of Diabetes. Diabetes Care 2017;40(Suppl 1):S11-s24.
26. Chobanian AV, Bakris GL, Black HR, et al. The Seventh Report of the Joint National Committee on Prevention, Detection, Evaluation, and Treatment of High Blood Pressure: the JNC 7 report. *Jama* 2003;289(19):2560-72.
27. Harrison R. Structure and function of xanthine oxidoreductase: where are we now? *Free Radic Biol Med* 2002;33(6):774-97.
28. Puig F, Rico F, Almendros I, et al. Vibration enhances interleukin-8 release in a cell model of snoring-induced airway inflammation. *Sleep* 2005;28(10):1312-6.
29. Real JT, Martínez-Hervás S, García-García AB, et al. Circulating mononuclear cells nuclear factor-kappa B activity, plasma xanthine oxidase, and low grade inflammatory markers in adult patients with familial hypercholesterolaemia. *Eur J Clin Invest* 2010;40(2):89-94.
30. Zanetti M, Zenti M, Barazzoni R, et al. HELP LDL apheresis reduces plasma pentraxin 3 in familial hypercholesterolemia. *PLoS One* 2014;9(7):e101290.
31. Ma J, Zhang H, Wang H, et al. Association Between Self-Reported Snoring and Metabolic Syndrome: A Systematic Review and Meta-Analysis. *Front Neurol* 2020;11:517120.
32. Zhang N, Chen Y, Chen S, et al. Self-Reported Snoring Is Associated with Dyslipidemia, High Total Cholesterol, and High Low-Density Lipoprotein Cholesterol in Obesity: A Cross-Sectional Study from a Rural Area of China. *Int J Environ Res Public Health* 2017;14(1)
33. Dai N, Shi Q, Hua Y, et al. Snoring frequency and risk of type 2 diabetes mellitus: a prospective cohort study. *BMJ Open* 2021;11(5):e042469.
34. Lee SK, Choi K, Chang YH, et al. Increased risk for new-onset hypertension in midlife male snorers: The 14-year follow-up study. *J Sleep Res* 2019;28(5):e12757.
35. Chang HJ, Lin KR, Lin MT, et al. Associations Between Lifestyle Factors and Reduced Kidney Function in US Older Adults: NHANES 1999-2016. *Int J Public Health* 2021;66:1603966.
36. Cui LF, Shi HJ, Wu SL, et al. Association of serum uric acid and risk of hypertension in adults: a prospective study of Kailuan Corporation cohort. *Clin Rheumatol* 2017;36(5):1103-10.
37. Peppard PE, Ward NR, Morrell MJ. The impact of obesity on oxygen desaturation during sleep-disordered breathing. *Am J Respir Crit Care Med* 2009;180(8):788-93.

For peer review only - http://bmjopen.bmj.com/site/about/guidelines.xhtml

38. Qi D, Liu J, Wang C, et al. Sex-specific differences in the prevalence of and risk factors for hyperuricemia among a low-income population in China: a cross-sectional study. *Postgrad Med* 2020;132(6):559-67.

39. Chen Y, Xia Y, Han X, et al. Association between serum uric acid and atrial fibrillation: a cross-sectional community-based study in China. *BMJ Open* 2017;7(12):e019037.

40. Qiu L, Cheng XQ, Wu J, et al. Prevalence of hyperuricemia and its related risk factors in healthy adults from Northern and Northeastern Chinese provinces. *BMC Public Health* 2013;13:664.

41. Jennum P, Hein HO, Suadicani P, et al. Cardiovascular risk factors in snorers. A cross-sectional study of 3,323 men aged 54 to 74 years: the Copenhagen Male Study. *Chest* 1992;102(5):1371-6.

For peer review only

For peer review only - http://bmjopen.bmj.com/site/about/guidelines.xhtml

Figure 1 Data cleaning flow chart

For peer review only

For peer review only - http://bmjopen.bmj.com/site/about/guidelines.xhtml

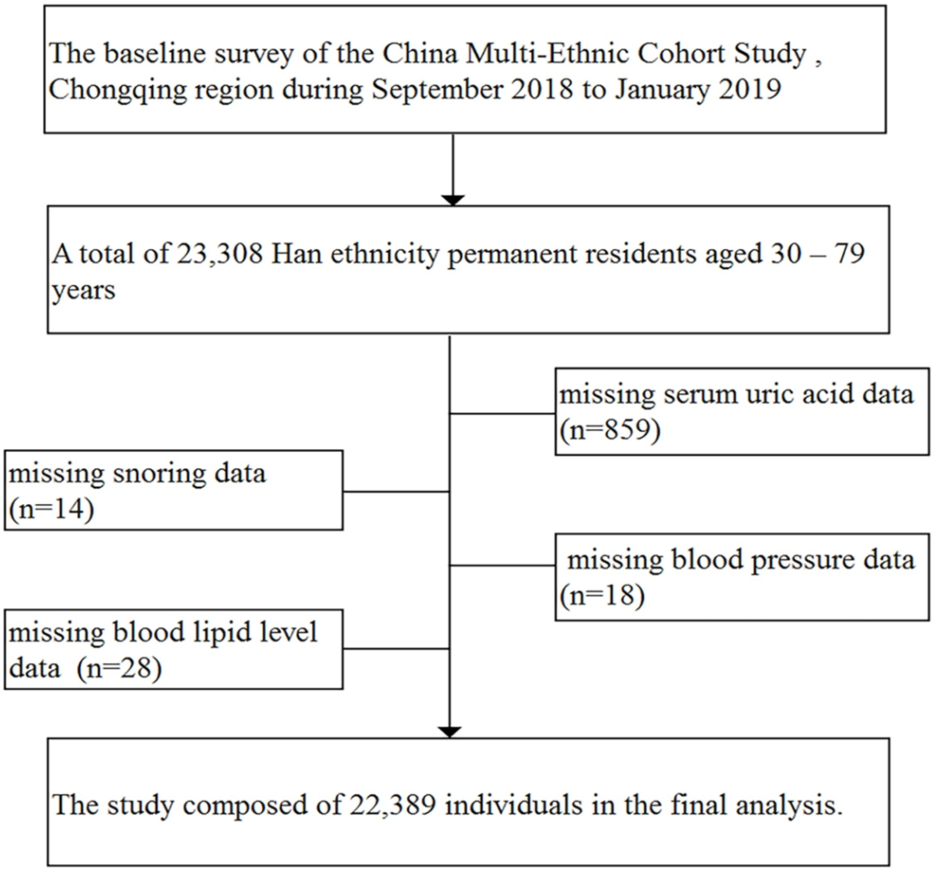

Figure 1 Data cleaning flow chart

101x101mm (300 x 300 DPI)

STROBE Statement—Checklist of items that should be included in reports of ***cross-sectional studies***

	Item No	Recommendation	Page No
**Title and abstract**	1	(*a*) Indicate the study's design with a commonly used term in the title or the abstract	1
		(*b*) Provide in the abstract an informative and balanced summary of what was done and what was found	2-3
**Introduction**			
Background/rationale	2	Explain the scientific background and rationale for the investigation being reported	3-4
Objectives	3	State specific objectives, including any prespecified hypotheses	4
**Methods**			
Study design	4	Present key elements of study design early in the paper	4
Setting	5	Describe the setting, locations, and relevant dates, including periods of recruitment, exposure, follow-up, and data collection	4-5
Participants	6	(*a*) Give the eligibility criteria, and the sources and methods of selection of participants	5
Variables	7	Clearly define all outcomes, exposures, predictors, potential confounders, and effect modifiers. Give diagnostic criteria, if applicable	5-8
Data sources/ measurement	8*	For each variable of interest, give sources of data and details of methods of assessment (measurement). Describe comparability of assessment methods if there is more than one group	5-8
Bias	9	Describe any efforts to address potential sources of bias	4
Study size	10	Explain how the study size was arrived at	5
Quantitative variables	11	Explain how quantitative variables were handled in the analyses. If applicable, describe which groupings were chosen and why	5-8
Statistical methods	12	(*a*) Describe all statistical methods, including those used to control for confounding	8
		(*b*) Describe any methods used to examine subgroups and interactions	8
		(*c*) Explain how missing data were addressed	5
		(*d*) If applicable, describe analytical methods taking account of sampling strategy	
		(*e*) Describe any sensitivity analyses	
**Results**			
Participants	13*	(a) Report numbers of individuals at each stage of study—eg numbers potentially eligible, examined for eligibility, confirmed eligible, included in the study, completing follow-up, and analysed	5,9
		(b) Give reasons for non-participation at each stage	5
		(c) Consider use of a flow diagram	5
Descriptive data	14*	(a) Give characteristics of study participants (eg demographic, clinical, social) and information on exposures and potential confounders	9-11
		(b) Indicate number of participants with missing data for each variable of interest	
Outcome data	15*	Report numbers of outcome events or summary measures	9
Main results	16	(*a*) Give unadjusted estimates and, if applicable, confounder-adjusted estimates and their precision (eg, 95% confidence interval). Make clear which confounders were adjusted for and why they were included	11-14

For peer review only - http://bmjopen.bmj.com/site/about/guidelines.xhtml

		(*b*) Report category boundaries when continuous variables were categorized	5-8
		(*c*) If relevant, consider translating estimates of relative risk into absolute risk for a meaningful time period	
Other analyses	17	Report other analyses done—eg analyses of subgroups and interactions, and sensitivity analyses	11-14
**Discussion**			
Key results	18	Summarise key results with reference to study objectives	14
Limitations	19	Discuss limitations of the study, taking into account sources of potential bias or imprecision. Discuss both direction and magnitude of any potential bias	18
Interpretation	20	Give a cautious overall interpretation of results considering objectives, limitations, multiplicity of analyses, results from similar studies, and other relevant evidence	14-17
Generalisability	21	Discuss the generalisability (external validity) of the study results	18
**Other information**			
Funding	22	Give the source of funding and the role of the funders for the present study and, if applicable, for the original study on which the present article is based	20

*Give information separately for exposed and unexposed groups.

Note: An Explanation and Elaboration article discusses each checklist item and gives methodological background and published examples of transparent reporting. The STROBE checklist is best used in conjunction with this article (freely available on the Web sites of PLoS Medicine at http://www.plosmedicine.org/, Annals of Internal Medicine at http://www.annals.org/, and Epidemiology at http://www.epidem.com/). Information on the STROBE Initiative is available at www.strobe-statement.org.

For peer review only

For peer review only - http://bmjopen.bmj.com/site/about/guidelines.xhtml
